# An emergent constraint on the thermal sensitivity of photosynthesis and greenness in the high latitude northern forests

**DOI:** 10.1038/s41598-024-56362-1

**Published:** 2024-03-14

**Authors:** Junjie Liu, Paul O. Wennberg

**Affiliations:** 1 Jet Propulsion Laboratory, California Institute of Technology, Pasadena, USA; 2 California Institute of Technology, Pasadena, USA

**Keywords:** Thermal sensitivity, Photosynthesis, High latitude northern forests, Emergent constraint, Climate sciences, Biogeochemistry, Carbon cycle

## Abstract

Despite the general consensus that the warming over the high latitudes northern forests (HLNF) has led to enhanced photosynthetic activity and contributed to the greening trend, isolating the impact of temperature increase on photosynthesis and greenness has been difficult due to the concurring influence of the CO_2_ fertilization effect. Here, using an ensemble of simulations from biogeochemical models that have contributed to the Trends in Net Land Atmosphere Carbon Exchange project (TRENDY), we identify an emergent relationship between the simulation of the climate-driven temporal changes in both gross primary productivity (GPP) and greenness (Leaf Area Index, LAI) and the model’s spatial sensitivity of these quantities to growing-season (GS) temperature. Combined with spatially-resolved observations of LAI and GPP, we estimate that GS-LAI and GS-GPP increase by 17.0 ± 2.4% and 24.0 ± 3.0% per degree of warming, respectively. The observationally-derived sensitivities of LAI and GPP to temperature are about 40% and 71% higher, respectively, than the mean of the ensemble of simulations from TRENDY, primarily due to the model underestimation of the sensitivity of light use efficiency to temperature. We estimate that the regional mean GS-GPP increased 28.2 ± 5.1% between 1983–1986 and 2013–2016, much larger than the 5.8 ± 1.4% increase from the CO_2_ fertilization effect implied by Wenzel et al. This suggests that warming, not CO_2_ fertilization, is primarily responsible for the observed dramatic changes in the HLNF biosphere over the last century.

## Introduction

Temperature over the northern hemisphere (NH) high latitudes (> 50° N) has been increasing at more than twice the rate of the rest of the globe. Given the continuing increases in greenhouse gases, this trend is unlikely to slow in the foreseeable future (IPCC AR6). Concurrently, ground and satellite observations have illustrated dramatic changes in terrestrial biosphere activity: longer growing season^1,2^, a greening trend over the majority of the region^3,4–7^, and an increase in carbon uptake from the atmosphere leading to an enhancement in the atmospheric CO_2_ seasonal cycle amplitude^8,9–12^. These trends alter photosynthesis, a process that converts light energy into chemical energy through electrochemistry fixing atmospheric carbon into organic carbon compounds through carboxylation^13^. The maximum rate of both electron transport and carboxylation increases exponentially with temperature before reaching an optimal temperature^14,15^. The mean growing season temperature over land > 50° N is between 5 and 18 °C (Fig. S1), generally lower than the optimal growth temperature for most plants even accounting for acclimation and adaptation^16,17^. Thus, the increase in temperature has been proposed as a mechanism that drives the increase of photosynthesis over the region^3,8, 9^. However, diagnosing the extent to which temperature enhances plants growth is complicated by the co-occurring increase in CO_2_ that enhances photosynthesis by increasing the difference in the rate of transport of CO_2_ and water through the stomata and increasing the efficiency of the carboxylating enzyme in C_3_ plants^18,19^, the so called “*CO*_*2*_* fertilization*” effect^20^.

The CO_2_ fertilization and warming effect on photosynthesis are relatively well-understood at leaf and canopy scale^15^, but there remains significant uncertainty in predictions of how these changes are altering the global carbon cycle. Consequently, the current state-of-science terrestrial biogeochemical models (TBMs) show a large range of the response of photosynthesis to climate change and CO_2_ increase^21,24^. For the HLNF, the uncertainty in the simulated net carbon uptake is close to 100%^22^.

A few past studies derived empirical emergent relationships between observables and model simulations of the carbon-climate feedback factor $\gamma$ over the tropics^23–25^, the photosynthesis-concentration feedback factor ${\beta }_{GPP}$ over the northern hemisphere (NH) mid to high latitudes^27^, and then used observations to constrain $\gamma$ and ${\beta }_{GPP}$ over these regions. Winkler et al.^26^ applied a similar concept to constrain the combined ${\gamma }_{GPP}$ and ${\beta }_{GPP}$ effect over the northern high latitudes. However, the quantitative impact of temperature increases on plant growth over the high latitudes (the ${\gamma }_{GPP}$ effect) is still elusive and is anticipated to be distinct from that in the tropics due to the large climatological differences^27^. Over the warm tropics, increasing temperature can reduce the terrestrial biosphere net carbon uptake from the atmosphere, causing positive carbon-climate feedbacks^23^, while over the high latitudes, the warming trend generally invigorates plants growth, enhancing CO_2_ uptake and thereby acting as a negative feedback to the climate^27^. The projections of future climate change critically depend on the understanding of these carbon-climate feedbacks. Here, we denote the photosynthesis-CO_2_-concentration feedback factor as ${\beta }_{GPP}$ and photosynthesis-climate feedback as ${\gamma }_{GPP}$ to distinguish them from the carbon-climate feedback factor $\gamma$ that describes changes of carbon pools due to climate^28^.

In this study, we expose an emergent relationship in the high latitude northern forest (HLNF) between the *spatially-derived* sensitivity of photosynthesis and greenness to temperature and the *temporal* changes driven by the changing climate from an ensemble of TBMs. Using the observed *spatial* sensitivity of both photosynthesis and greenness, we constrain how photosynthesis and greenness are responding to warming ${(\gamma }_{GPP})$ that has occurred over the past decades (Fig. S1). Our approach is complementary to the tropical carbon-climate feedback factor proposed by Cox et al.^23^ and Sullivan et al.^24^, the ${\beta }_{GPP}$ effect on GPP over temperate and boreal forest derived by Wenzel et al.^29^, and the combined ${\gamma }_{GPP}$ and ${\beta }_{GPP}$ effect over the high latitudes by Winkler et al.^26^. The emergent relationship derived here is based on our earlier study^10^ which introduced the use of the *spatial* sensitivity of photosynthetic activity to temperature to infer historical *temporal* changes in photosynthesis. As the spatial gradient in CO_2_ is both transient in nature (due to atmospheric transport) and always small (generally less than 15 parts per million (ppm)), the CO_2_ effect on the *spatial* sensitivity of GPP and greenness to temperature is negligible. The spatial sensitivity of GPP and greenness to temperature reflects the potential equilibrium sensitivity of vegetation to warming that includes the effect of real-world physiological and ecological adaption^24^, thus it is suitable to infer long-term sensitivities of vegetation to warming.

## Results

### Spatially-derived sensitivity of GPP and greenness to temperature

The emergent constraint on the warming effect on plants growth derived in this study builds upon the relationships between *spatial* sensitivity of GPP and greenness to temperature and their corresponding *temporally-derived* sensitivities to temperature. Thus, we first quantify the spatial sensitivity of GPP and greenness to temperature (Fig. S1 and sections “Materials and methods”, “Workflow to derive the observation-constrained photosynthesis/greenness: climate feedback factors”). As shown in Liu et al.^10^ (Fig. S5), the observed spatially-derived sensitivity of greenness to temperature over the HLNF is time-invariant. We anticipate that if the *spatially-derived* sensitivity of GPP and greenness to temperature from models is also time-invariant, then the simulated *temporal* changes of GPP and greenness caused by temperature would be similar to that predicted by the corresponding spatial sensitivity to temperature. Thus, we first evaluated the temporal consistency of the simulated spatial sensitivities of GPP and greenness to temperature in current terrestrial biogeochemical models. We examined both Leaf Area Index (LAI) and GPP from an ensemble of TBMs from the Trends in Net Land Atmosphere Carbon Exchange project (TRENDY) v6, and simulations where only CO_2_ was varied (S1) and simulations where both CO_2_ and climate were varied (S2). The differences between these two runs reflect the impact of climate change only. LAI is generally defined as one-half of the total green leaf area per unit horizontal ground surface area with unit of m^2^/m^2^^30^; GPP is a function of both the absorption of photosynthetically active radiation (APAR) (related to greenness) and light use efficiency (LUE), which is a function of many factors including environmental drivers, e.g., temperature^31^. APAR is a product of photosynthetic radiation (PAR) and the fraction of absorbed PAR (fPAR) by plants. As fPAR and LAI are interchangeable through Beer’s law approximation^32^, we estimate fPAR from the LAI reported from the TRENDY models to disentangle the contributions of both greenness and LUE to the sensitivity of GPP to temperature.

Since the S1 runs are driven by a 20 years repeating climatology, we calculated 20 years mean GPP and LAI from the TRENDY models starting at 1901. To increase the sample size, we subsampled these into 10-year overlap (e.g., 1901–1920, 1911–1930, etc.), which results in 10 groups each for S1 and S2 runs for each model. We selected grid cells (> 50° N) with at least 40% tree cover fraction (Table S1), and then fitted the correlation between growing season mean temperature (GS-T) and growing season GPP and LAI for each group from each model using an exponential fit (section “Materials and methods”). We selected 40% as a forest threshold to remove grid cells with dominant grassland and cropland vegetation types in both model runs and observations, since water availability could be dominant climate driver over these vegetation types^33^.

**Figure 1:**
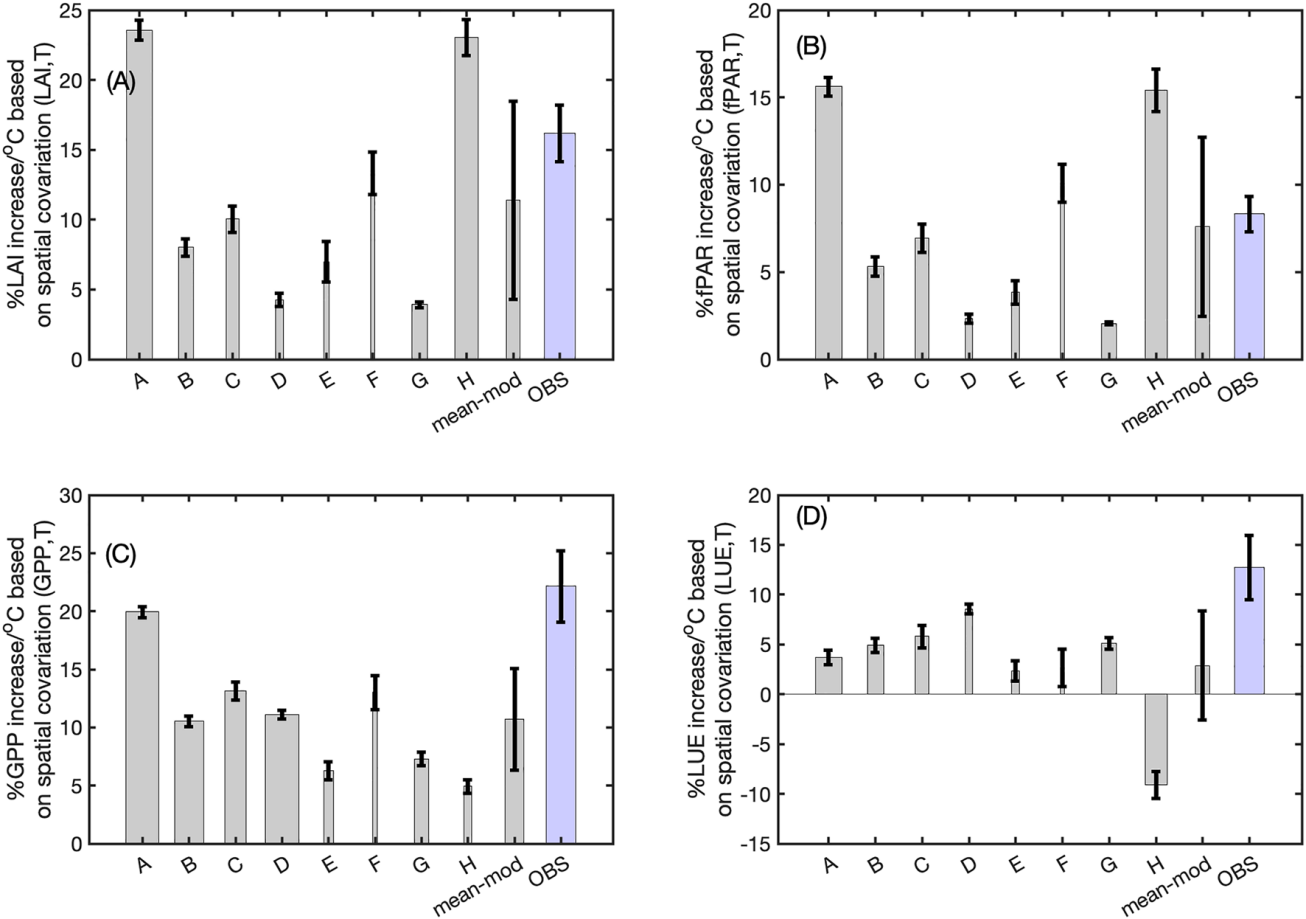
The spatially-derived sensitivity of growing season LAI and GPP to temperature does not change over the 100 year simulations by TRENDY. The ensemble model mean underestimates the GPP sensitivity diagnosed from observations, primarily coming from underestimation in light use efficiency (LUE) not fPAR. (**A**) The spatially-derived sensitivity of growing season LAI to temperature for the TRENDY S2 ensemble models (A–H), model mean (mean-mod), and the LAI (OBS). (**B**) The spatially-derived sensitivity of growing season fPAR to temperature from the TRENDY S2 ensemble models (A–H), model mean (mean-mod), and the observations (OBS). The fPAR for TRENDY models were calculated from LAI following the Beer’s law approximation^32^. (**C**) The spatially-derived sensitivity of growing season GPP to temperature for the TRENDY S2 ensemble models (A–H), model mean (mean-mod), and the observations (OBS). (D) The spatially-derived sensitivity of growing season LUE to temperature for the TRENDY S2 ensemble models (A–H), model mean (mean-mod), and the observations (OBS). The unites are %/°C for all these quantities. The model names corresponding to each model ID is listed in Table S1. The width of the bars represents how well correlated LAI and GPP are with temperature (the wider the bar, the higher the correlation; see details in Table S1).

An exponential, rather than linear relationship, is used following the general description of the dependence of both electron transport and carboxylation on temperature in cold ecosystems, such as those analyzed here^14,15^ (section “Materials and methods”). Farquar et al.^15^ shows that the carbon assimilation rate follows a nonlinear curve before reaching an optimal temperature. The same study shows that the temperature dependence of the kinetic properties of rubisco carbonxylase rate follows an exponential relationship. Furthermore, using a linear model the limiting behavior at low temperature is pathological: the implied photosynthetic rate would be negative even at temperatures above freezing (Fig. S3). The exponential fitting also allows us to calculate the Q10 values for both GPP and LAI, which indicates fractional changes of GPP and LAI per 10 °C increase in temperature. The Q10 concept is broadly used in describing the relationship between soil respiration and temperature^34^. The R^2^ (GPP or LAI, T) shows how strongly the spatial distributions of GS- GPP and LAI are coupled to GS-T in each model. The standard deviation of the exponential fitting coefficients among the 10 groups in each model shows the degree of time-invariance in the spatial sensitivity of GPP and LAI to GS-T with time. The exponential fitting implies that the spatial sensitivity of GS- GPP or LAI to GS-T can be expressed as the percentage change of GPP and LAI per 1 °C *spatial* gradient in GS-T, which is a function of the exponential fitting coefficients (Fig. S1, section “Carbon-climate feedback factors from TRENDY models” in section “Materials and methods”). We used GPP constrained by Solar Induced Chlorophyll Fluorescence (SIF) from Orbiting Carbon Observatory -2 (OCO-2)^10,34^, FLUXCOM GPP products^35^, and four LAI and fPAR products to calculate the spatial sensitivity of the observed GPP, LAI, and fPAR to temperature (section “Materials and methods”) (Figs. S3–S5). We calculated the spatial sensitivity of LUE to temperature as the difference between the spatial sensitivity of GPP to temperature and the spatial sensitivity of fPAR to temperature, since GPP can be written as a product of APAR and LUE. While observations show a strong spatial coherence between GS-T and GPP, LAI, fPAR, and LUE (R^2^ values of at least 0.6; Figs. S3–S5), the simulations in both S1 and S2 runs show a large range in the strength of the correlation between the spatial GS-T and GPP_,_ LAI_,_ or fPAR (R^2^ values ranges from 0 to 0.7; Fig. 1, Table S1, and Figs. S7–S8). In the subsequent analysis, we only used those models with R^2^ larger than 0.1 for either LAI-T or GPP-T relationship. We further required that the model outputs include monthly LAI and GPP. The spatial distributions of GS-GPP or GS-LAI from the excluded models (I, J, and K) have almost no correlation with GS-T (Fig. S9). In total, there are eight models that pass the criteria (Table S1). We refer these eight remaining models as “*TRENDY models*” in later discussion.

Despite the large variations in the strength of the correlation, the magnitude of the spatial sensitivity of GS- GPP and LAI to GS-T are largely time-invariant in both the S1 and S2 runs in all models (Figs. 1 and S6). For example, the spatial sensitivity of GS- LAI to GS-T from model A varies by less than 10% in the decadal averages of the 100-year simulations (22.8%/°C to 24.2%/°C) (Fig. 1). For each model, the spatially-derived sensitivity of GPP and LAI to temperature is similar between S1 and S2 runs (Figs. 1, S6), indicating that these characteristics are likely intrinsic to the construction of each model. The S3 runs, which adds to S2 the process of land use land cover change, have a similar spatially-derived sensitivity to temperature as S1 and S2 runs (Fig. S6). As the growing season varies across latitudes (Fig. S10), there is almost no co-variation between GS-T and GS-PAR (Fig. S11).

The TRENDY models underestimate the spatial sensitivity of both GPP and LAI to temperature compared to the observations (Figs. 1, S7–S8). The sensitivity of fPAR to temperature, while highly variable across the models, span the observed sensitivity (Fig. 1). The mean sensitivity of GS- LAI to GS-T from the TRENDY S2 runs ($${P}_{LAI}^{G}$$ in Fig. S1) is 11.6 ± 7.2%/°C, ~ 30% lower than the sensitivity of the observed LAI to temperature (16.2 ± 2.0%/°C). The mean sensitivity of GS-fPAR to GS-T is 7.6 ± 5.2%/°C, almost the same as the observed GS-fPAR to GS-T sensitivity (8.3 ± 1.0%/°C), while the mean spatially-derived sensitivity of GPP to temperature ($${P}_{GPP}^{G})$$ is 10.7 ± 4.4%/°C, only half that observed (22.1 ± 3.0%/°C). The fact that the TRENDY models significantly underestimate the sensitivity of GPP to temperature but have comparable sensitivity of fPAR to temperature implies that the TRENDY models significantly underestimate the sensitivity of LUE to temperature over the NH high latitude forests (Figure 1D), consistent with the conclusion of Thomas et al.^32^. Model H even has negative LUE sensitivity to temperature, while simulates much larger spatial sensitivity of LAI to temperature than the observed. All the eight models underestimate the spatial sensitivity of GPP to temperature, with model A closest to the observed value. However, model A assigns the majority of the sensitivity to greenness, while still significantly underestimates the LUE sensitivity to temperature. The partitioning of changes in GPP between APAR and LUE is important as it may contribute to the timescale of the dynamics of these forests. For example, increases in APAR due to earlier start of the growing season may be both fast and large - the forest can respond quickly to warmer springtimes (when PAR is at its seasonal maximum) with simply earlier leafout. In contrast, changes in LUE that arise from structural changes in the forest (e.g. the growth of trees and their rooting depth, changes in nutrient dynamics, or, in particular, succession) would be expected to have much longer timescales for response.

Using the same tree cover fraction map from MODIS for all the models, the mean spatial sensitivity of LAI to temperature in the TRENDY models slightly increased from 11.6 ± 7.2 to 14.2 ± 5.2%/°C compared to when the model-specific tree cover fraction map was used (Figs. S12–S14). The difference arises mainly from models D and G which define all land cover north of 66° N as tundra or grasslands. The mean spatial sensitives of GPP to GS-T are almost the same when using the same MODIS tree cover among all models compared to when model-specific tree cover map was used (12.0 ± 4.3 vs. 10.7 ± 4.4%/°C).

### Emergent constraint on temporal sensitivity of GPP and LAI temperature

The fact that the spatial sensitivity to temperature is nearly time-invariant in the TRENDY simulations (Figs. 1, S6) implies that absent other changes, the historical LAI and GPP would simply shift to higher values as temperature increases following the relationship defined by the spatially-derived sensitivity. This indicates that the sensitivity derived from the observed spatial dependence of GPP and LAI on temperature can be used as an emergent constraint on how warming has driven the changes in GPP and LAI historically.

**Figure 2:**
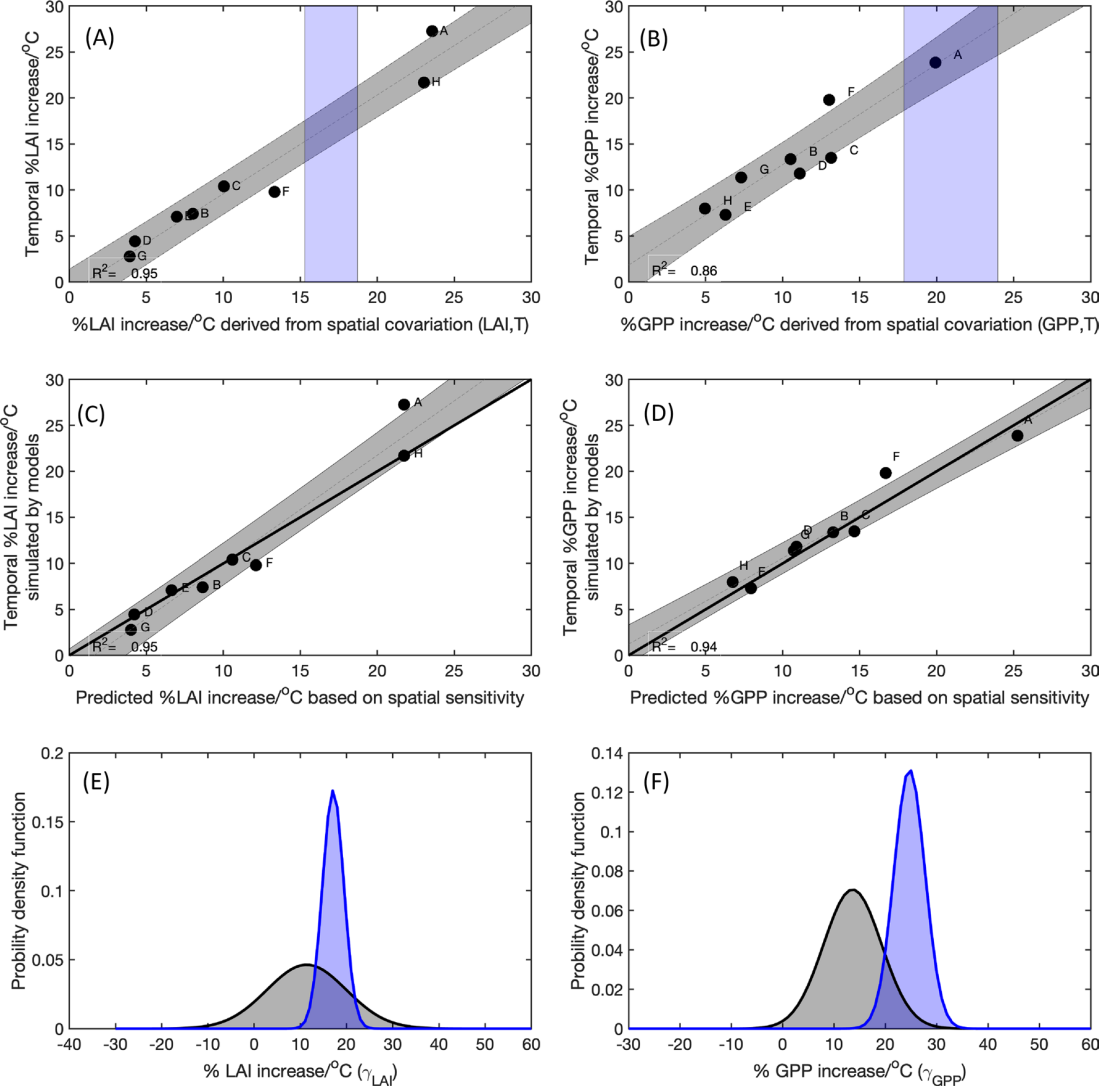
An emergent constraint on the percentage changes of LAI and GPP due to warming since 1901 with the corresponding spatially-derived sensitivity to temperature observed over the high latitude northern forests. (**A**) The relationship between the spatially-derived and the temporally-derived sensitivities of LAI to temperature in the simulations (${\gamma }_{LAI}^{G}$). (**B**) The relationship between the spatially-derived and temporally-derived sensitivity of GPP to temperature in the simulations (${\gamma }_{GPP}^{G}$). The blue shaded area in A and B shows the observational-derived spatial sensitivity. (**C**) and (**D**) The relationship between the actual model simulated temporal sensitivity (y-axis) and the predicted sensitivity based on the corresponding spatially-derived sensitivity from each model. Blue line is 1:1 line, and the dashed grey line is the best linear fitting line. (**E**) The unconstrained probability density function distribution of ${\gamma }_{LAI}^{G}$ across models (grey bars), which assumes that all TRENDY models have equal possibility and that their distribution is Gaussian. The blue area represents the conditional probability distribution derived by applying the observation constraint (blue shaded area in **A**) to the across-model relationship. (**F**) The unconstrained probability density function distribution of ${\gamma }_{GPP}^{G}$ across models (grey bars), which assumes that all of the TRENDY models have equal possibility and their distribution is Gaussian. The blue area represents the conditional probability distribution derived by applying the observation constraint (blue shaded area in B) from B to the across-model relationship. The unconstrained model mean ${\gamma }_{LAI}^{G}$ is 12.2 ± 8.3%/°C (1 $\sigma$), and the constrained ${\gamma }_{LAI}^{OBS}$ is 17.0 ± 2.4%/°C. The emergent constraint reduces the uncertainty in ${\gamma }_{LAI}^{G}$ by 72%. The unconstrained ${\gamma }_{GPP}^{G}$ is 14.0 ± 6.0%/°C the constrained ${\gamma }_{GPP}^{obs}$ is 24.0 ± 3.0%/°C. The emergent constraint reduces the uncertainty in ${\gamma }_{GPP}^{G}$ by 50%.

Figure 2A,B shows the spatial sensitivity of GPP and LAI, respectively, to temperature vs. the corresponding temporal sensitivity to temperature during the growing season (section “Spatial sensitivity of growing season mean GPP and LAI to the growing season mean temperature” in section “Materials and methods”). The models with larger spatial sensitivity to temperature predict larger percentage increase in GPP and LAI with increasing temperature. Furthermore, in each model, the temporal change in GPP and LAI predicted from the spatial sensitivity (section “Materials and methods”) agrees well with the simulated temporal sensitivity between 1901 and 2010 (Fig. 2C,D). Figure 2C,D shows that seven out of the eight models lie on the 1:1 line in the comparison between the predicted temporal sensitivity and the model simulated temporal sensitivity for both GPP and LAI. The large R^2^ values (0.94 and 0.86 respectively) between the spatially-derived sensitivity and the temporal sensitivity, and the predictability of temporal sensitivity using the corresponding spatial-derived sensitivity within TRENDY models provide compelling evidence that the observed spatial sensitivity of GPP and LAI to temperature provides an emergent constraint on the photosynthetic-climate feedback factor ${\gamma }_{GPP}$ or ${\gamma }_{LAI}$ over the HLNF.

Based on the emergent relationship between the spatial sensitivity and temporal sensitivity derived from the TRENDY models (Fig. 2A,B) and the constraint provided by the observed spatially-derived sensitivity of GPP and LAI to temperature, we calculated that LAI increased 17.0 ± 2.4%/°C (${\gamma }_{LAI}^{obs})$ and GPP increased 24.0 ± 3.0%/°C (${\gamma }_{GPP}^{obs})$, which translate to ${Q}_{10}^{LAI}$ and ${Q}_{10}^{GPP}$ to be 1.7 ± 0.24 and 2.4 ± 0.3 respectively. These values are about 40% and 71% higher than the TRENDY model mean, which are 12.2 ± 8.3% (${\gamma }_{LAI}^{G}$) and 14.0 ± 6.0%/°C (${\gamma }_{LAI}^{G}$) respectively (Fig. 2C,F). The TRENDY model mean attributes GPP and LAI increase to temperature and CO_2_ effect almost equally. When accounting for both climate and CO_2_ effect, the mean temporal sensitivities of the TRENDY models are 19.8 ± 10.9%/°C and 27.1 ± 8.0%/°C for LAI and GPP respectively (section “Materials and methods, Figs. S19–S20), only slightly higher than the observationally constrained sensitivity to temperature alone. Among the eight models, model A has the smallest CO_2_ effect (near 0—Figs. S19–S20), also has a spatial sensitivity to temperature most similar to observations.

Using the same tree cover fraction map defined by MODIS in all models, the emergent constraint implies that LAI increased 13.5 ± 3.0%/°C (Fig. S17). The somewhat lower temporal sensitivity is due to the time invariant LAI simulations in models D and G over grassland and tundra north of 66° N as defined in these two models (Figs. S15–S16), which changes the slope between the spatial sensitivity and temporal sensitivity. However, the predicted GPP temporal sensitivity using the MODIS tree cover fraction is similar (23.0 ± 4.0%/°C) (Fig. S17). The significant underestimation of the sensitivity of plant growth to temperature over the high latitudes may have contributed to the weaker carbon sink simulated by these models^12,36^ and, subsequently, the much smaller CO_2_ seasonal cycle amplitude changes attributed to climate by TRENDY models (Fig. S18, section “Materials and methods”).

The emergent constraint reduces the uncertainty of estimated LAI and GPP temporal sensitivity by 72% and 50%, respectively. The uncertainty of emergent constraint includes uncertainties in the observed spatial sensitivity to temperature, the linear fitting between the spatially-derived sensitivity to temperature and the temporally-derived sensitivity to temperature (section “Materials and methods”). The smaller uncertainty reduction in the estimated sensitivity of GPP to changing temperature is primarily due to the larger uncertainty (compared to LAI) in the observed spatial sensitivity of GPP. As discussed in Winkler et al.^26^, the emergent constraint method is particularly sensitive to the uncertainties in the observed quantity. In calculating the probability function for the models, we assume equal weights for each model.

The observed carbon climate feedback factor ${\gamma }_{GPP}^{obs}$ is much larger than the magnitude of ${\gamma }_{LAI}^{obs},$ because GPP is a function of both greenness and LUE. In cold ecosystems without water limitation, LUE increases with temperature exponentially^10^, contributing to the additional sensitivity of GPP to temperature beyond the sensitivity of greenness to temperature. The fact that the ${\gamma }_{GPP}^{G}$ has similar magnitude as ${\gamma }_{LAI}^{G}$ in the TRENDY models indicates these models primarily underestimate the sensitivity of light use efficiency to temperature, consistent with Fig. 1 and the conclusion of Thomas et al.^37^ We hope that the emergent constraint described here motivates model developers to simulate and report the spatial dependence of GS-LAI or GS-GPP on GS-T. The lack of these fields in models I–K excluded them from our analysis; it is also the reason that model F is an outlier in Fig. 2 (Figs. S7–S8).

### Contributions of warming only to the historical LAI and GPP increase over the northern high latitude forests

**Figure 3:**
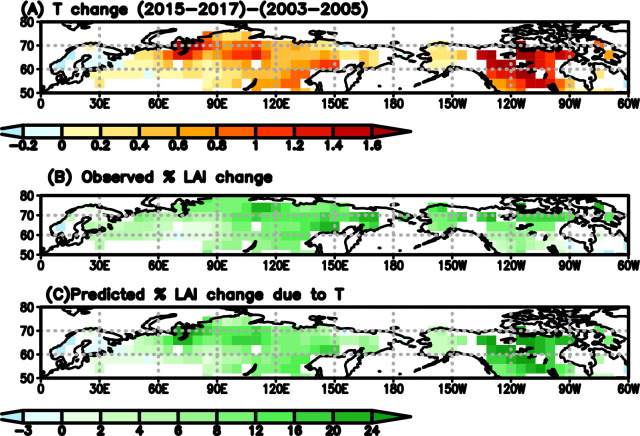
The predicted percentage change in LAI due to temperature change have similar spatial pattern and magnitude as observations. (**A**) The growing season mean temperature difference between 2003–2005 and 2015–2017. (**B**) The mean percentage change in LAI derived from MCD-MODIS, GIMMS-MODIS, and GIMMS-AVHRR. (**C**) The predicted percentage change in LAI between 2003–2005 and 2015–2017 calculated from ${\gamma }_{LAI}^{obs} .$ (temperature data is derived from ERA-5 reanalysis).

To evaluate ${\gamma }_{LAI}^{obs}$ and quantify the contributions of temperature increase to the LAI and GPP increase over the region, we predicted growing season LAI and GPP changes using ${\gamma }_{LAI}^{obs}$ and ${\gamma }_{GPP}^{obs}$, and then compared these to the observed changes. We made predictions over two time intervals: between the period 2003–2005 and 2015–2017 when MODIS observations are available, and between the period 1983–1986 and 2013–2016 to quantify the long-term changes in LAI and GPP and compared these to the changes driven by CO_2_ fertilization based on Wenzel et al.^29^. The predicted percentage change of LAI due to the increase in growing-season temperature from the spatial sensitivity have similar spatial pattern and magnitude as the observations, especially over Eurasia (Fig. 3). The predicated mean LAI increase between 2003–2005 and 2015–2017 over Eurasia is 7.6% ± 1.1% during growing season, very close to the observed mean increase (8.3 ± 1.7%) averaged over the three LAI products: MCD-MODIS, GIMMS-AVHRR, and GIMSS-MODIS. The predicted LAI change over North America (NA) is somewhat larger than the observed value (14.7 ± 2.0% vs. 8.2 ± 2.0%), perhaps reflecting land use change and disturbance that is not accounted here^38^.

Between 1983–1986 and 2013–2016, the GS-T increases more than 0.4 °C over most of the Eurasia and northern NA (Fig. S21). The observationally-constrained photosynthesis-climate feedback factors ${\gamma }_{LAI}^{obs}$ and ${\gamma }_{GPP}^{obs}$ implies that the warming contributes to a 9–27% increase in LAI and a 12–35% increase in GPP over most of the region (Fig. S21B). On average, the regional mean growing-season LAI increases 18.9 ± 2.8% based on the ${\gamma }_{LAI}^{obs}$, comparable to 19.6% increase in the NOAA AVHRR LAI product, but somewhat larger than the GIMMS-AVHRR LAI product (14.2%). The discrepancy with GIMMS-AVHRR is again over NA, while over Eurasia, the predicted GS-LAI increase (17.8 ± 2.6%) is very similar to the observed LAI increase (17.3%). The regional mean GPP increases 28.2 ± 5.1% based on ${\gamma }_{GPP}^{obs}$, which is much larger than the impact of CO_2_ fertilization. Based on Wenzel et al.^29^, the GPP increases 5.8 ± 1.4% over the high latitude as a result of the 54.2 parts per million (ppm) CO_2_ increase during this time period (section “Materials and methods”). The combination of the temperature effect estimated in this study and the CO_2_ effect based on Wenzel et al.^29^ indicates that the mean of TRENDY models significantly overestimate the CO_2_ effect and underestimate the temperature effect on the historical increase of GPP and LAI.

## Discussion

Two pieces of empirical evidence, TRENDY model simulations (Fig. 2C,D) and the comparison between LAI predictions and observations (Fig. 3), support the validity of constraining the temporal sensitivity of growing-season photosynthesis to temperature with its spatial sensitivity to temperature over the HLNF. For the space-for-time substitution to work, one of the challenges is to ensure that similar physical and biological processes govern both the spatial and temporal variations. We argue that the spatial sensitivity of photosynthesis to temperature over high latitude northern forests reflects close-to-equilibrium response to temperature variations, and thus to a large extent it is suitable to predict the long-term temporal sensitivity^24^. The sensitivity curves derived from the spatial co-variations between temperature and photosynthesis not only include the temperature effect on nutrient availability, greenness, light use efficiency, but also possible species succession with temperature increases. All these aspects are qualitatively supported by observations. Studies have shown that low latitudes with higher temperature has higher soil net nitrogen mineralization and simultaneously soil net nitrogen mineralization increases with temperature^39^, supporting that spatial and temporal variability of nutrient availability share similar features. The spatial sensitivity curve (Figs. S3–S4) uses data from all forest types over high latitudes (Fig. 2 in Liu et al.^10^). Thus, the temporal prediction using the spatial sensitivity also includes possible species succession with temperature increases, which is supported by limited field measurements^40^. The species migration with temperature increase implied by the spatial sensitivity curve implies that it may be possible to develop adaptive management strategies to accommodate shifts in species ranges, ensuring the preservation of biodiversity and resilience of forest ecosystems to climate change stressors in the future. Because the spatial sensitivity curve includes temperature effect on species succession that takes decades, the spatial sensitivity curve may overpredict the temperature impact on the interannual variations of productivity.

The emergent constraint described in this study indicates that the TRENDY models underestimate the impact of historical temperature increases on greenness and GPP over the HLNF, primarily due to the underestimation of the sensitivity of light use efficiency to temperature. The fact that the mean temporal sensitivities of the TRENDY models that account for both temperature and CO_2_ effect are much closer to the observation-constraint temperature-only sensitivity indicate that the major weakness in these models is the attribution of the total changes to specific processes. The apparent discrepancy between model simulated spatial sensitivity of GPP and LAI to GS-T with the corresponding observation-derived quantities implies deficiencies of these models in simulating temperature effect on plants growth over HLNF. Together with Wenzel et al.^29^, our results resolve the partition of the competing effect between CO_2_ fertilization and warming over the HLNF, providing a unique metric to improve process representation in the models and reduce uncertainties in quantifying photosynthesis-climate feedback factors over the HLNF. While the dominant warming effect on enhancing plants growth over high latitudes identified in our study is consistent with factorial model simulations described by Forkel et al.^8^ and Winkler et al.^41^, our study provides independent evidence based on direct observational constraint.

Our study shows that the increase in temperature is the dominant factor that has driven the GPP increase and the historical greening trend over HLNF. As temperature over the HLNF continues to increase at much higher rate than the rest of the globe, it is critical to monitoring how greenness and photosynthesis are changing and to be on alert to any tipping point where increasing temperatures no longer act as negative feedbacks to climate^42,43^.

## Materials and methods

### Workflow to derive the observation-constrained photosynthesis/greenness: climate feedback factors

Figure S1 describes the workflow we used to derive the observation-constrained photosynthesis/greenness—climate feedback factors ${\gamma }_{GPP}^{obs}$ and ${\gamma }_{LAI}^{obs}$ , which are then used to predict changes of GPP and LAI from historical increase in temperature over the high latitude northern forests. We derived the photosynthesis/greenness—climate feedback factors ${\gamma }_{GPP}^{obs}$ and ${\gamma }_{LAI}^{obs}$ in three steps. First, we fitted spatial covariation of growing season GPP and LAI with growing season mean temperature for both the Trends in Net Land Atmosphere Carbon Exchange project (TRENDY) model runs and observations, and calculated the spatial sensitivity of GPP and LAI to temperature for both TRENDY models ${P}^{G}$ and observations ${P}^{obs}$ (sections “Materials and methods”, “Growing season definition”). Second, we caclulated the historical GPP and LAI change due to climate change only from TRENDY, and fitted a functional curve to derive temporal sensitivity of GPP and LAI change to temperature change ${\gamma }^{G}$ (sections “Materials and methods”, “Spatial sensitivity of growing season mean GPP and LAI to the growing season mean temperature”). Third, we derived an emergent relationship between the TRENDY spatial sensitivity ${P}^{G}$ and the temporal sensitivity ${\gamma }^{G}$ to temperature, and constrain the photosynthesis/greenness—climate feedback factors ${\gamma }_{GPP}^{obs}$ and ${\gamma }_{LAI}^{obs}$ using the emergent relationship derived in the second step and the observed spatial sensitivity ${P}^{obs}$ derived in the first step (Fig. S1).

### Growing season definition

In each of the 20-year group of the S1 and S2 runs, we first calculated the monthly climatology of GPP from each TRENDY model. Based on the monthly climatology in each time span, we defined the growing season at each model grid point as the time period when GPP is larger than 20% of the maximum *GPP* at that grid. We chose a 20% threshold to reduce the impact of errors of the observational-constrained GPP at low values (section “Results”). We only chose those grids that have well-defined spring (20% to 80% of maximum GPP before maximum GPP) and fall season (20% to 80% of maximum GPP after maximum GPP) that have at least three samples to calculate growing season mean value. Lastly, we chose those grids that have at least 40% tree cover and have valid definition of growing season throughout the whole time period (Table S1). Note that the grids for each model are the same set of grid points throughout the time period, but could differ among the models. The length of the growing season can also change, and the type of trees may change at any selected grid during the study time period. As shown in Figs. S7–S8, the GS-T varies across models and latitudes, which is due to the large uncertainties in phenology simulations over high latitudes^44^.

### Spatial sensitivity of growing season mean GPP and LAI to the growing season mean temperature

In this study, we used two types of GPP products and four LAI products to calculate the observationally-constrained spatial sensitivity of GPP and LAI to temperature, which is defined as the percentage change of GPP and LAI per 1°C spatial gradient in the GS-T. The GPP products include OCO-2 solar induced chlorophyll fluorescence (SIF) constrained GPP^10^ and the FluxCom GPP products that are based on three different neural network algorithms^45^. To derive the observationally-constrained spatial sensitivity, we fitted an exponential function between the spatial distribution of growing season GPP or LAI and growing season T (GS-T) for each product (Fig. S3). The exponential form is chosen as the maximum rate of both electron transport and carboxylation increases exponentially with temperature before an optimal temperature for plants growth is reached. The exponential form can be written as:

1 $$C_{i} = aexp\left( {d \cdot T_{i} } \right)$$

where *C*_*i*_ can be either GPP or LAI at grid point *i*, *a* and *d* are fitting coefficients and *T*_*i*_ is the growing season mean temperature GS-T at grid point *i*. Then, the fraction change of *C* in space can be written as:

2 $$\frac{{C_{i} }}{{C_{0} }} = exp\left( {d \cdot (T_{i} - T_{0} )} \right)$$

Then the observationally-constrained spatial sensitivity of GPP and LAI to temperature ${P}_{o}$ (Fig. S1) can be written as:

3 $$P_{o} = \left( {exp\left( d \right) - 1} \right) \times 100$$

The SIF-constrained GPP gives the highest sensitivity (27 ± 2%/°C). The three different FluxCom GPP products have the sensitivities of 23 ± 2, 21 ± 2, and 16 ± 2%/°C. The mean spatial sensitivity of GPP to temperature across the four products is 22.1 ± 3.0%/°C, which is used here to define the observationally-constrained spatial sensitivity of GPP to temperature in Fig. 2. The uncertainty includes the uncertainty in both the exponential fitting and the standard deviation among the four products.

The large spatial sensitivity of GPP to temperature is not due to the co-variation of temperature and photosynthetic active radiation (PAR). As shown in Fig. S11, the spatial distribution of growing season PAR (GS-PAR) has no relationship with spatial distribution of temperature (GS-T). This is because the growing season starts later over the higher latitudes, when the solar radiation becomes stronger (Fig. S10), so that the mean GS-PAR varies little across the high latitudes.

We chose exponential fitting instead of linear fitting because the exponential is more consistent with photosynthetic theory. Farquar et al.^15^ shows that the carbon assimilation rate follows a nonlinear curve before reaching an optimal temperature. The same study shows that the temperature dependence of the kinetic properties of rubisco carbonxylase rate follows exponential relationship. Furthermore, the limiting behavior at low temperature is pathological using a linear model: the implied photosynthetic rate would be negative even at temperatures above freezing. As implied by the exponential fitting curve, the linear fitting slope would depend on the mean temperature.

We used four different LAI products: MCD-MODIS, GIMMS LAI3g^46^, GIMMS MODIS-LAI^47^, and NOAA AVHRR. Following the same procedure used to analyze GPP, we fitted an exponential function between the spatial distribution of growing season LAI and GS-T for each model product (Fig. S4). The two AVHRR products have similar spatially-derived sensitivity of LAI to GS-T (~ 15 ± 1%/°C), and the two MODIS LAI products have the spatially-derived sensitivity of 20 ± 2 m^2^/m^2^/°C and 15 ± 1%/°C, respectively. The mean spatially-derived sensitivity of LAI to temperature is 16.2 ± 2%/°C and is used as our observationally-constrained sensitivity of LAI to temperature.

We regridded all the data to 4° × 5° to reduce sampling errors, especially the SIF-constrained GPP products. But as shown in Liu et al.^10^, the spatial sensitivity is similar between 1° × 1° and 4° × 5° resolution.

### Carbon-climate feedback factors from TRENDY models

To calculate the carbon-climate feedback factor at each grid cell, we assumed that the temporal relationship of GPP and LAI with temperature at every grid cell *i* follows a similar exponential function as their spatial relationships with temperature, but with the possibility of different coefficients from the spatially-derived sensitivity:

4 $$(g_{n,i} ) = g_{1,i} \exp \left( {b(T_{n,i} - T_{1,i} )} \right)$$

$\mathrm{The symbols }{g}_{n}$ and ${g}_{1}$ represent growing season GPP or LAI at time group *t*_*n*_ and *t*_1_, and ${T}_{n}$ and ${T}_{1}$ represent growing season *T* at time group *t*_*n*_ and *t*_1,_ respectively. Based on Eq. (4), we define:

5 $$\Delta g_{n,i} = log\left( {\frac{{g_{n,i} }}{{g_{1,i} }}} \right) = b\left( {T_{n,i} - T_{1,i} } \right)$$

To calculate carbon-climate feedback factor $\gamma$ from TRENDY simulations, we used both S1 and S2 runs. The S1 runs have time-varying CO_2_ concentrations, but repeat the 1901–1920 climate every 20 years. The S2 runs have time-varying CO_2_ and climate. As the difference between S1 and S2 is whether they have time-varying climate, we can isolate the impact of climate using these two experiments. We calculated growing season mean GPP or LAI every 20 years with 10-year overlap from 1901 to 2010 to remove the impact of time-varying *T* on S1 runs and to ensure a large enough sample size. In total, we have 10 groups, so *n* is from 2 to 10.

In the following, we derive the calculation of carbon-climate feedback factor $\gamma$. First, based on Eq. (5) we define ${\Delta g}_{(S2-S1)}$ as:

6 $$\Delta g_{{\left( {S2 - S1} \right)}} = \frac{1}{m}\left( {\mathop \sum \limits_{i = 1}^{m} {\text{log}}\left( {\frac{{g_{n,i} }}{{g_{1,i} }}} \right)_{S2} - \mathop \sum \limits_{i = 1}^{m} {\text{log}}\left( {\frac{{g_{n,i} }}{{g_{1,i} }}} \right)_{S1} } \right)$$

where *m* represents the total number of grid points. The Eq. (6) can be further written as

7 $$\Delta g_{( S2 - S1)} = \frac{1}{m}\left( {\mathop \sum \limits_{i = 1}^{m} \log \frac{(g_{n,i} )_{S2} }{(g_{n,i} )_{S1} } = (b_{S2} - b_{S1} } \right)\frac{1}{m}\mathop \sum \limits_{i = 1}^{m} (T_{n,i} - T_{1,i} )$$

From Eqs. (6) to (7), we assume that both S2 and S1 start from the same initial state, so ${{(g}_{1,i})}_{S2}={{(g}_{1,i})}_{S1}$. Note, Eq. (7) is still valid, even when S2 and S1 runs have different initial state, but the terms ${{(g}_{1,i})}_{S2}$ and ${{(g}_{1,i})}_{S1}$ cannot be canceled out. We define the fraction change of carbon state GPP or LAI ${\Delta C}_{(S2-S1)}$ and ${\Delta T}_{n-1}$ respectively as:

8 $$\Delta C_{{\left( {S2 - S1} \right)}} = \exp \left( {\Delta g_{{\left( {S2 - S1} \right)}} } \right) = \sqrt[m]{{\left( {\mathop \prod \limits_{i = 1}^{m} \frac{{(g_{n,i} )_{S2} }}{{(g_{n,i} )_{S1} }}} \right.}}$$

9 $$\Delta T_{{\left( {t_{n} - t_{1} } \right)}} = \frac{1}{m}\mathop \sum \limits_{i = 1}^{m} \left( {T_{n,i} - T_{1,i} } \right)$$

${\Delta C}_{(S2-S1)}$ represents the spatial mean ratio between *C* at time *t*_*n*_ and time *t*_*1*_. Any value other than one in ${\Delta C}_{(S2-S1)}$ indicates the impact of temperature only on changes of *C* as shown in Eq. (6). *C* can be either GPP or LAI. Then, Eq. (8) can be written as:

10 $$\Delta C_{{\left( {S2 - S1} \right)}} = {\text{exp}}\left( {(b_{S2} - b_{S1} )\Delta T_{{\left( {t_{n} - t_{1} } \right)}} } \right)$$

We fitted exponential curves between ${\Delta C}_{(S2-S1)}$ and ${\Delta T}_{({t}_{n}-{t}_{1})}$ for both GPP and LAI in Figs. S19 and S20. In Figs. S19 and S20, we also fitted exponential curves between ${\Delta C}_{(S2)}$ and ${\Delta T}_{({t}_{n}-{t}_{1})}$, which indicate changes of *C* due to both temperature and CO_2_ effect. Since the range of ${\Delta T}_{({t}_{n}-{t}_{1})}$ is small, the exponential curve is similar to a linear line.

Thus, the *fractional change* of GPP and LAI between time *t*_*n*_ and *t*_*1*_ is:

11 $$\Delta C_{{\left( {S2 - S1} \right)}} - 1 = {\text{exp}}\left( {((b_{S2} - b_{S1} )\Delta T_{{\left( {t_{n} - t_{1} } \right)}} } \right) - 1{ }$$

The percentage of GPP and LAI per 1 °C changes in time ${\gamma }^{G}$ for each model *G* can be written as:

12 $$\gamma^{G} = (\exp \left( {b_{S2 - S1} } \right) - 1) \times 100$$

where ${b}_{S2-S1}={(b}_{S2}-{b}_{S1})$, ${\gamma }^{G}$ is the photosynthesis-climate feedback factor from each model. ${b}_{S2-S1}$ is derived from the fitting shown in Figs. S19 and S20 and ${\gamma }^{G}$ is summarized in the y-axis in Fig. 2a–d.

### Prediction of temporal sensitivity with the spatially-derived sensitivity in TRENDY models

We calculated the temporal sensitivity $\Delta H$ with the spatially-derived sensitivity from each model using the following equation:

13 $$\Delta H = \frac{{\mathop \sum \nolimits_{i = 1}^{m} \left[ {\exp \left( {d_{S2} T_{n,i} } \right) - \exp \left( {d_{S2} T_{1,i} } \right)} \right]}}{{\mathop \sum \nolimits_{i = 1}^{m} \exp \left( {d_{S2} T_{1,i} } \right) }} \times 100$$

where ${d}_{S2}$ is the mean spatial sensitivity averaged over the 10 groups from each model, and ${T}_{n,i}$ and ${T}_{1,i}$ is the growing season mean temperature at the *i*th grid cell in the 10th (1991–2010) and 1^st^ (1901–1920) temporal group respectively (Fig. S1). $\Delta G$ is the value plotted on the x-axis on Fig. 2C,D.

### Calculation of the GPP increase between 2006–2015 and 1983–1992 due to CO_2_ increase based on Wenzel et al.^29^

We first calculated the mean annual CO_2_ concentration over these two time periods using the data available at https://gml.noaa.gov/webdata/ccgg/trends/co2/co2_annmean_gl.txt, and found that the mean CO_2_ concentration over these two time periods was 398.9 and 344.7 ppm, respectively. We then calculated the difference to get the CO_2_ change. As Wenzel et al.^29^ derived that the GPP over the high latitude (60–90°) increase 37 ± 9% with doubling of CO_2_ concentration and 32% ± 9% over the extratropics, we calculated the GPP increase due to the CO_2_ increase according to 37 × (398.9–344.7)/344.7 = 5.8. We calculated the uncertainty as 9 × (398.9–344.7)/344.78 = 1.4. Wenzel et al.^29^ accounted for all vegetation types, while in our study, we only quantified the impact of temperature change on forest growth.

### Calculation of CO_2_ seasonal cycle amplitude (SCA) change attributed to climate change over the NH high latitude forests by TRENDY models

We ran the GEOS-Chem atmospheric transport model with the net ecosystem exchange (NEE) from each of the selected TRENDY models. We only used the NEE over the forest regions between 50 and 75 N from either the S1 runs or S2 runs over 1958–1963 (IGY time period) or between 2009 and 2011 (HIPPO time period), and set the NEE to zero over the rest of the globe. For different transport model runs, we used the same meteorology fields between 2006 and 2011, with the extra years as a spin-up. We then sampled the simulated CO_2_ concentration fields along the IGY or HIPPO aircraft campaign tracks, and calculated the CO2 SCA at every 10° latitude interval following Liu et al.^10^ from each run. The mean differences of CO_2_ SCA change between S2 and S1 runs are the values plotted in Fig. S18, and the standard deviations are the uncertainties.

### Significance statement

The high latitude northern forests have experienced dramatic changes in recent decades including a general greening trend that has enhanced the atmospheric CO_2_ seasonal cycle amplitude. The increase in both temperature and atmospheric CO_2_ can contribute to such changes, making it challenging to partition the response of the HLNF to forcings from the change in climate and CO_2_. This challenge contributes to the large uncertainties in climate projections. Here we show using both ensemble model simulations and observations, that the sensitivity of gross primary productivity (GPP) and leaf area index (LAI) to temperature in space can predict their temporal changes due to warming, thereby isolating the temperature effect from CO_2_. We find that increasing temperature, not increasing CO_2_, is responsible for most of the trends in GPP and LAI over the past decades. In contrast, biogeochemical models generally assign 50% or more of the increase in GPP to CO_2_ fertilization.

### Supplementary Information

Supplementary Information.

## Data Availability

All data used to support the findings of this study are publicly available. TRENDY model simulations and its met drivers are available on request from TRENDY coordinator Dr. S. Sitch (s.a.sitch@exeter.ac.uk). The GIMMS AVHRR and MODIS LAI data are available upon request from by Dr. Ranga Myneni (rmyneni@bu.edu). The NOAA AVHRR LAI data is available at: https://www.ncei.noaa.gov/access/metadata/landing-page/bin/iso?id=gov.noaa.ncdc:C01559. The MCD-MODIS is available at: https://lpdaac.usgs.gov/products/mcd15a2hv006/. The FLUXCOM GPP dataset was obtained from https://www.bgc-jena.mpg.de/geodb/projects/Data.php. The OCO-2 SIF data is publicly available at https://disc.gsfc.nasa.gov/datasets/OCO2_L2_Lite_SIF_10r/summary?keywords=oco2%20sif%20lite The CERES data is available at https://asdc.larc.nasa.gov/project/CERES.
