# Supplementary material for: An emergent constraint on the thermal sensitivity of photosynthesis and greenness in the high latitude northern forests
Source: Sci Rep. 2024 Mar 14;14:6189. doi: 10.1038/s41598-024-56362-1 (PMC11319809; doi:10.1038/s41598-024-56362-1)
Supplement: Supplementary file 1 — Supplementary Information. [file 41598_2024_56362_MOESM1_ESM.pdf]

Supporting Information for

An emergent constraint on the thermal sensitivity of photosynthesis and greenness in the high latitude northern forests

Junjie Liu[1,2*] and Paul O. Wennberg[2*]

1. **Jet Propulsion Laboratory, California Institute of Technology, USA**
2. **California Institute of Technology, USA**

Email: junjie.liu@jpl.nasa.gov; wennberg@caltech.edu

This PDF file includes:

Figures S1 to S20

Tables S1

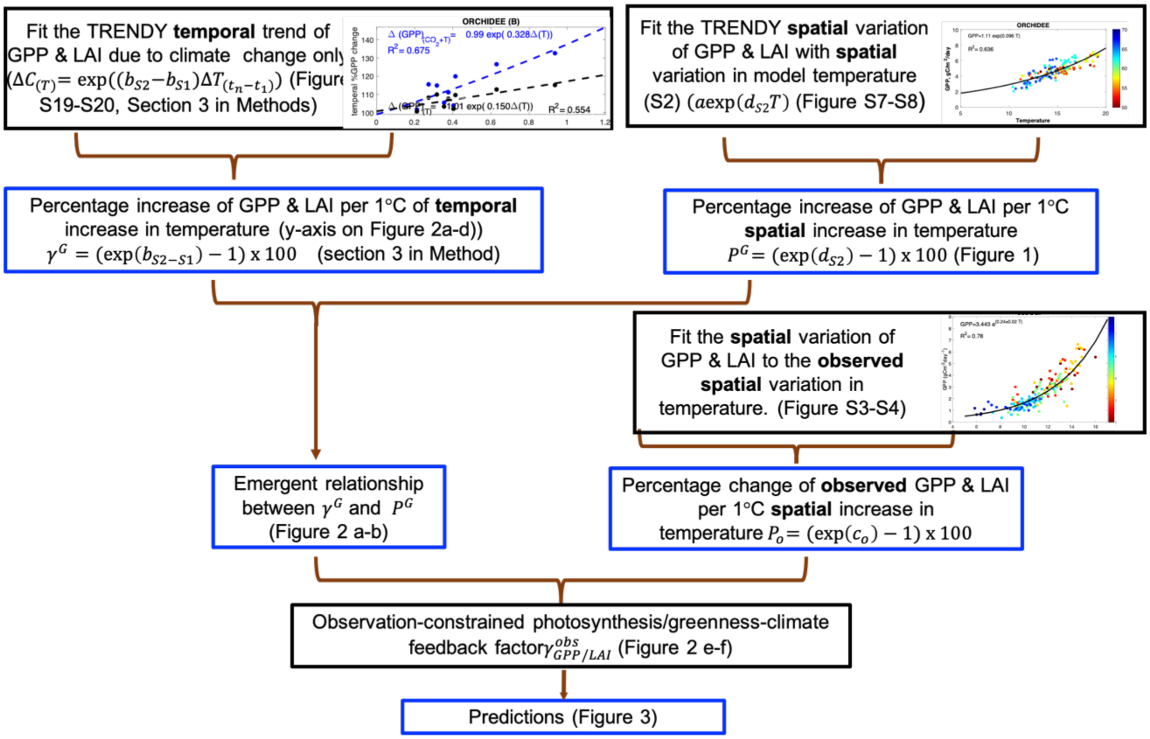

Figure S1 Workflow to derive the observation-constrained photosynthesis/greenness – climate feedback factors γ^{obs}_{GPP} and γ^{obs}_{LAI} , which are then used to predict changes of GPP and LAI from historical increase in temperature over the high latitude northern forests. S2 and S1 represent TRENDY S2 and S2 runs respectively. S2 runs have varying climate and CO_2, and S1 runs have varying CO_2.

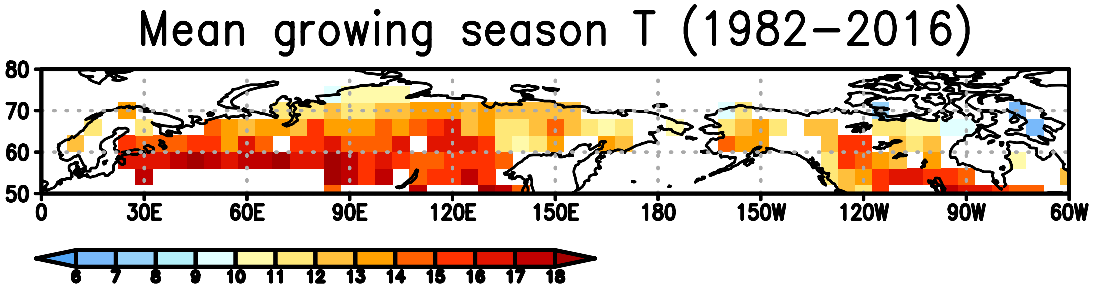

Figure S2 Mean growing season temperature between 1982 and 2016 (unit: °C) over grids (4° (latitude) x 5° (longitude)) with tree cover fraction larger than 40%.

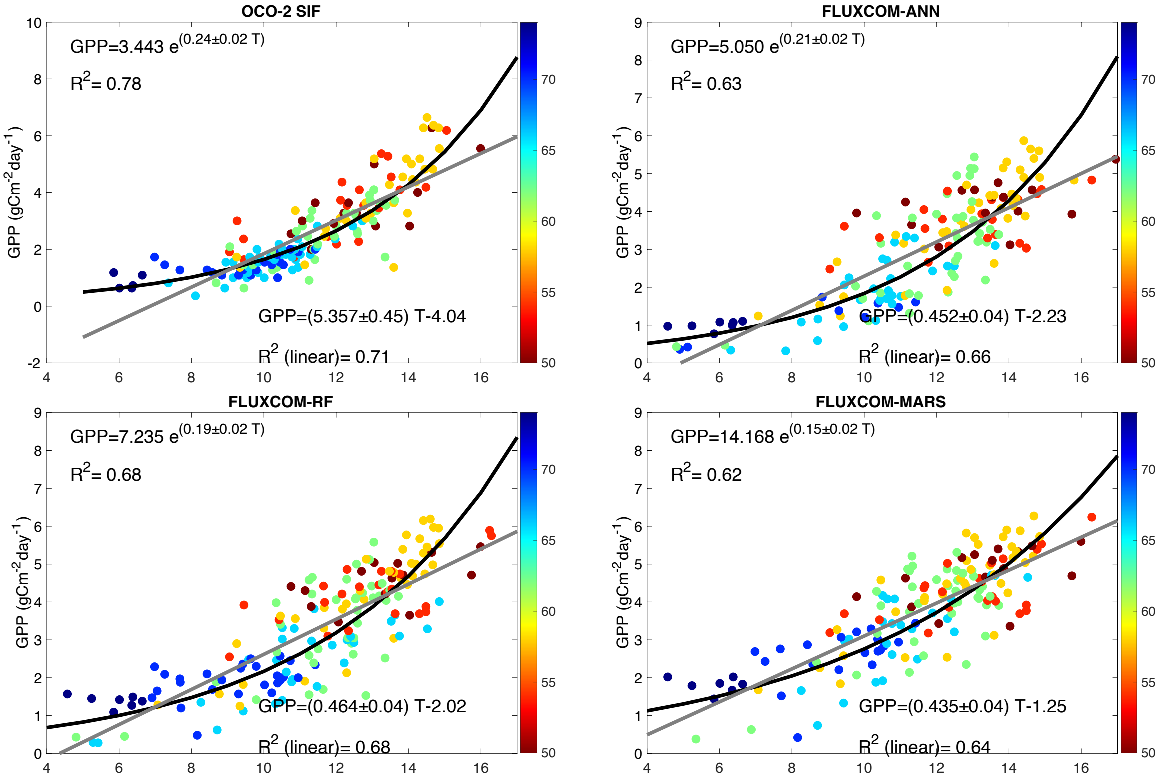

Figure S3 The spatially-derived relationship between GS- GPP and GS- temperature. The points include grid cells located north of 50 degrees latitude with at least 40% tree cover, with color-coded by latitudes. A. OCO-2 SIF-constrained GPP; B. FLUXCOM GPP based on ANN algorthm; C. FLUXCOM GPP based on random forest (RF); D. FLUXCOM GPP based on MARS algorithm. X-axis: temperature (unit, °C), and y-axis is GPP with unit of $gC/m^2/day$. The black curve shows exponential fitting between temperature and GPP while the gray line shows linear fitting.

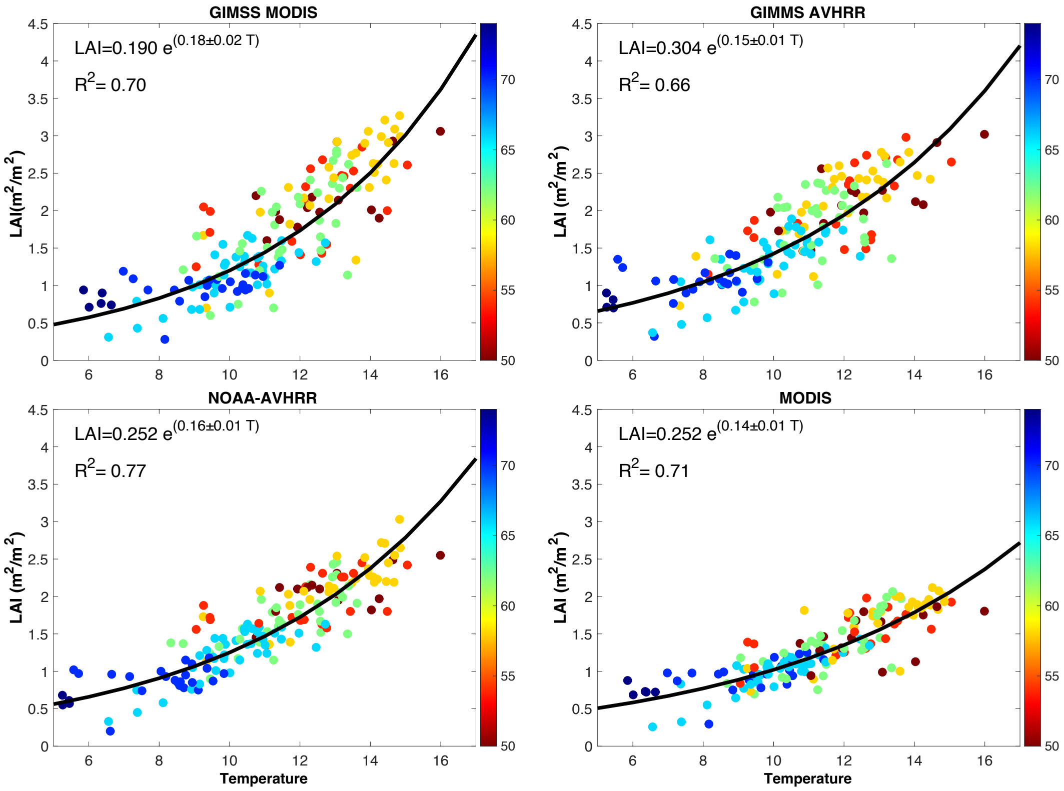

Figure S4 The spatially-derived relationship between GS- LAI to GS-temperature. The points include grid cells located north of 50 degrees latitude with at least 40% tree cover. The dots are color-coded by latitudes. A: GIMMS MODIS LAI; B. GIMSS AVHRR LAI; C. NOAA AVHRR LAI; D. MODIS LAI; X-axis: temperature (unit, °C), and y-axis is LAI with the unit (m^2/ m^2).

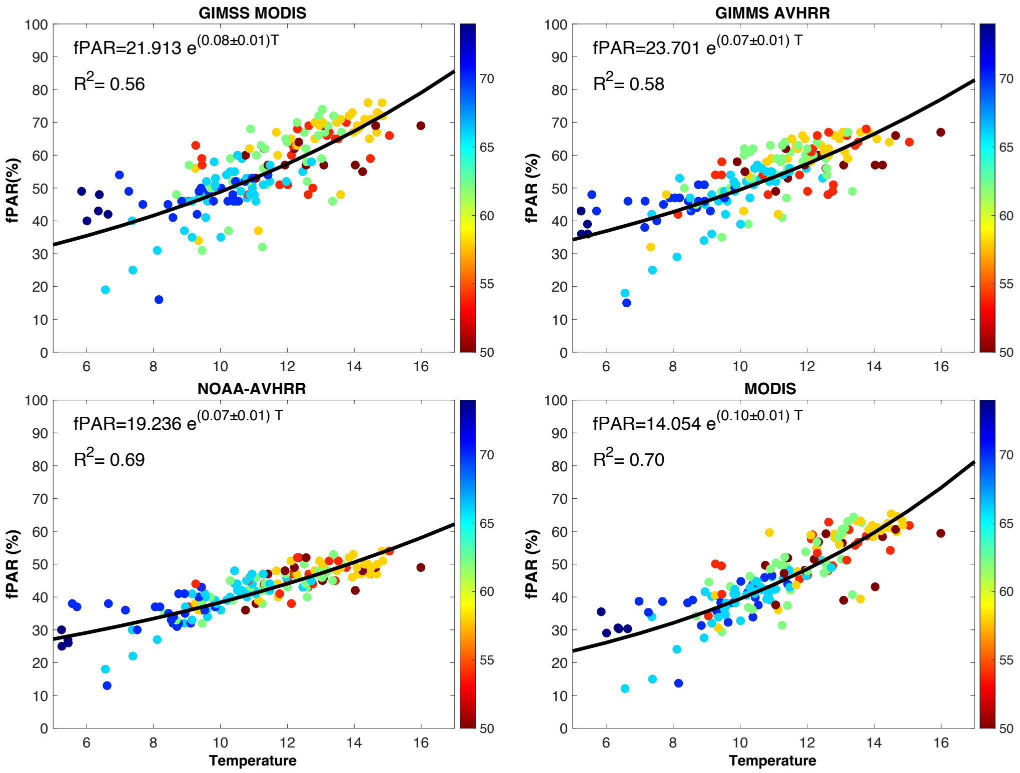

Figure S5 The spatially-derived relationship between GS- fPAR (fraction of Photosynthetic Active Radiation) and GS- temperature. The points include grid cells located north of 50 degrees latitude with at least 40% tree cover. The color bar represents latitudes of those points. A: GIMMS MODIS; B. GIMSS AVHRR; C. NOAA AVHRR; D. MODIS MCD15 product; X-axis: temperature (unit, °C), and y-axis is fPAR with the unit (%).

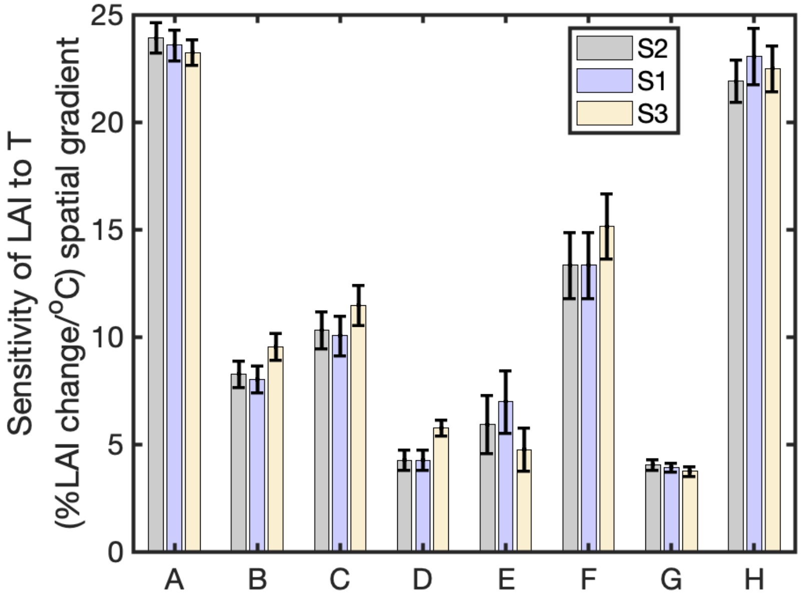

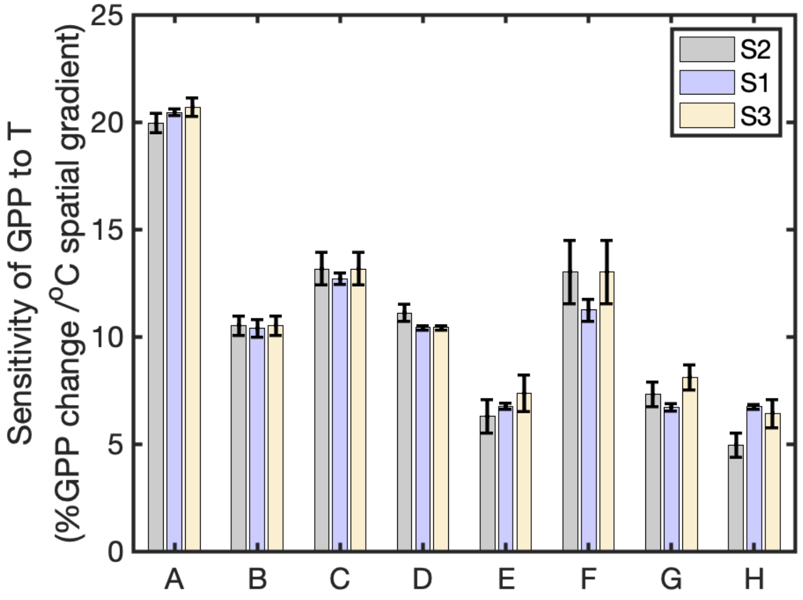

Figure S6 The S1, S2, and S3 runs have similar magnitude of spatially-derived sensitivity of growing season LAI (A) and GPP (B) to temperature. The error bars are the standard deviations of the corresponding spatially-derived sensitivity among the 10 groups in each model. The model names corresponding to each model ID are listed in Table S1.

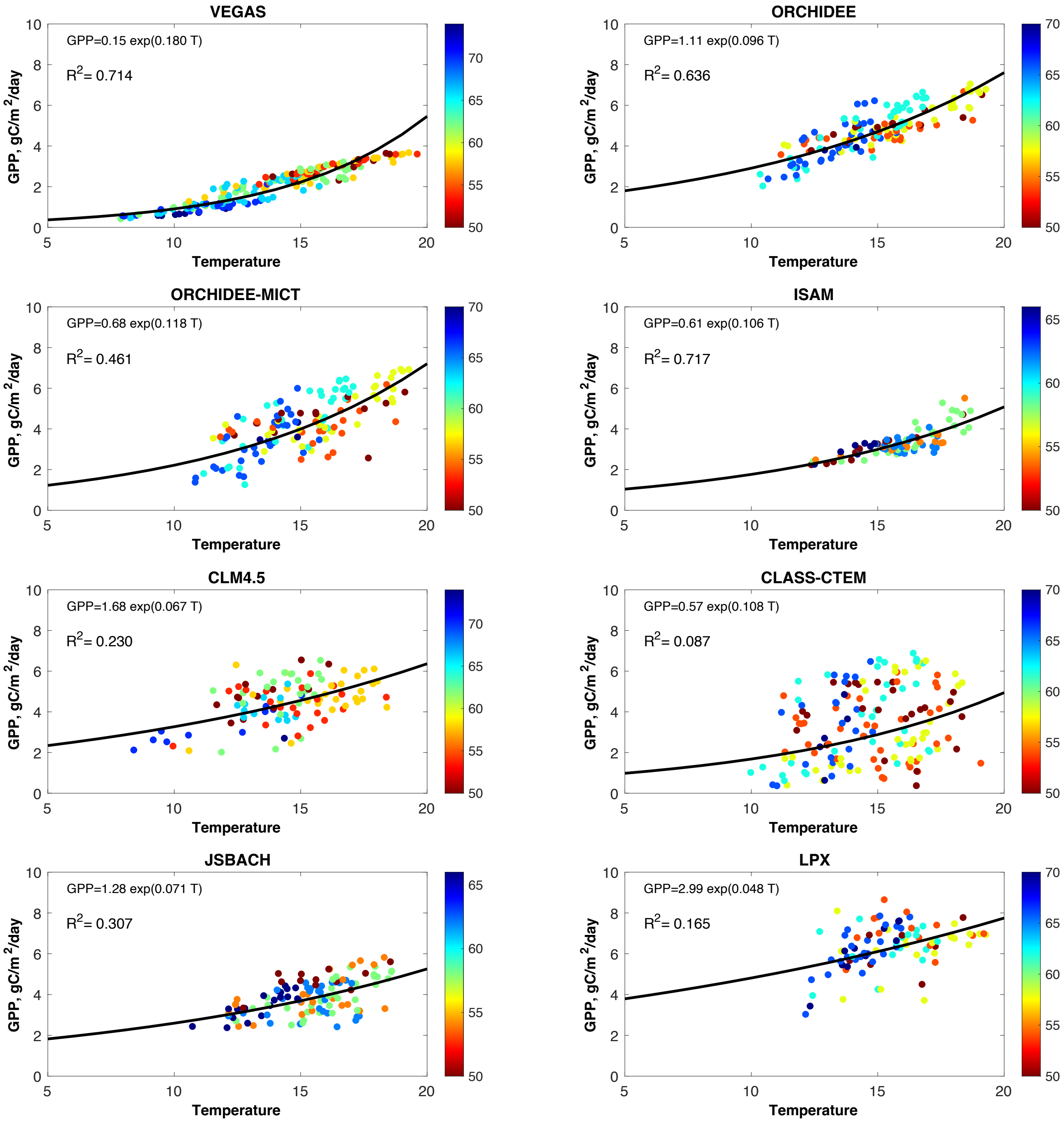

Figure S7 The spatial covariation between growing-season GPP (GS-GPP) and growing-season temperature during one of the 20-year groups (1971-1990). The points include grid cells located north of 50 degrees latitude with at least 40% tree cover listed in Table S1. The color bar represents latitudes of those points. On top of each panel is the name of TRENDY models.

1

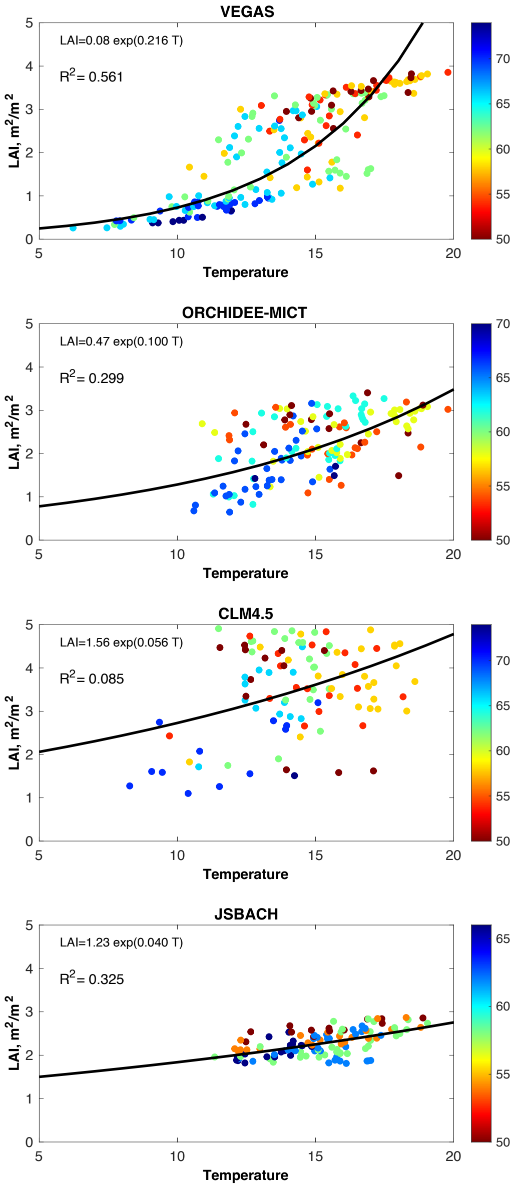

Figure S8 The same as Figure S7, except this is for LAI with unit of m^2/m^2.

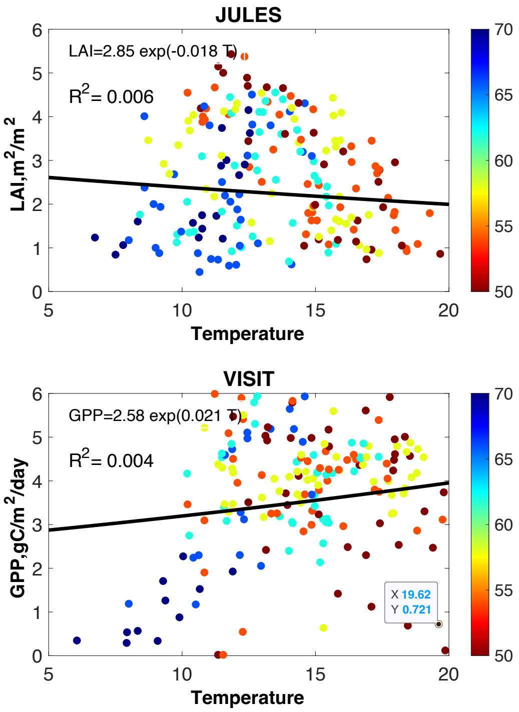

Figure S9 The spatial covariation between growing-season mean GPP (GS-GPP) or LAI to temperature during one of the 20-year groups (1971-1990) for the models that were not selected in this study due to its low spatial coherence with temperature spatial gradient. The points include grid cells located north of 50 degrees latitude with at least 40% tree cover listed in Table S1. The color bar represents latitudes of those points. On top of each panel is the name of TRENDY models.

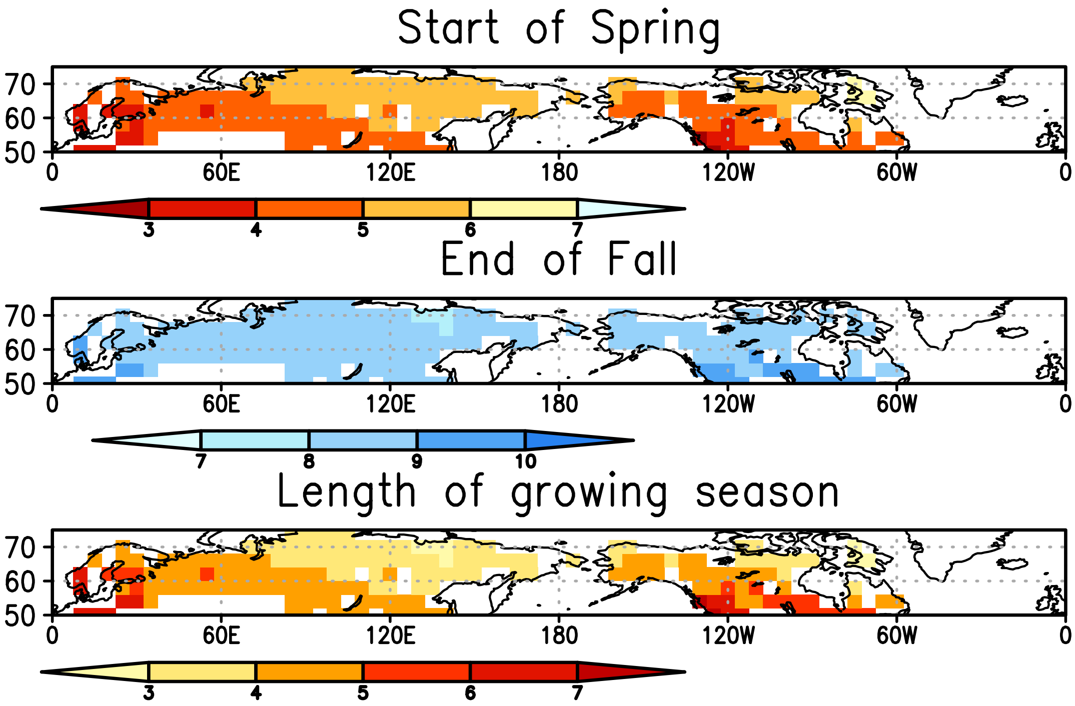

Figure S10 Growing season definition based on SIF-constrained GPP.

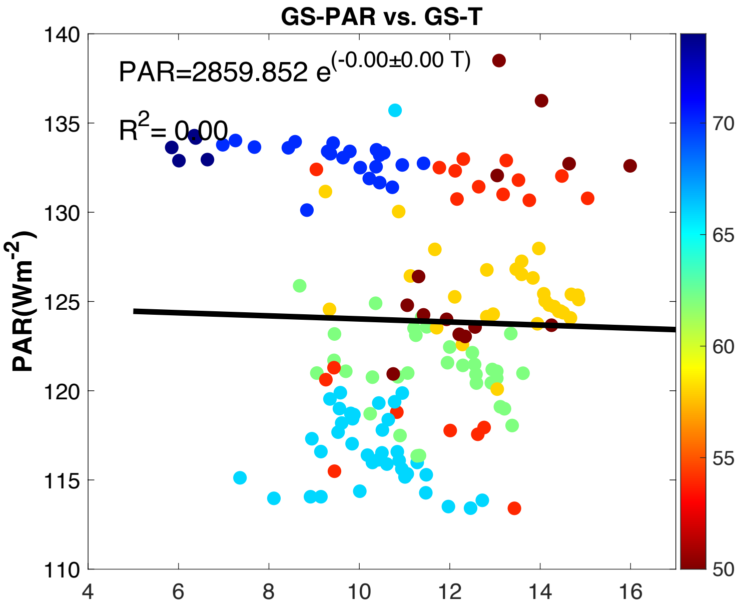

Figure S11 The relationship between growing season mean photosynthetic active radiation (PAR) (W/m^2) and growing season mean temperature (°C) colored by latitude (degrees).

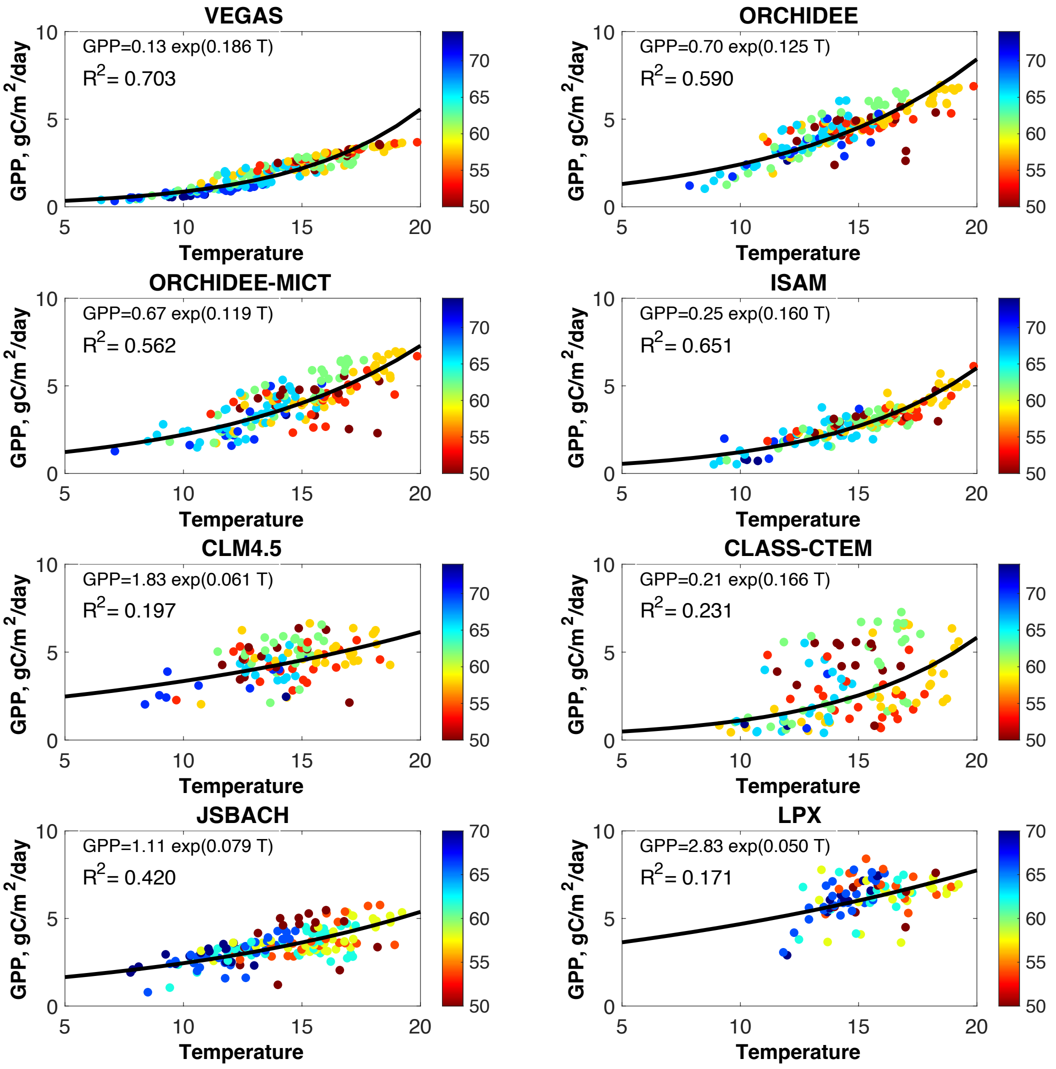

Figure S12 The same as Figure S7, except that all the models using the same condensed MODIS tree cover fraction. We have excluded grid points with near zero GS-GPP values and grid cells that do not have clearly-defined growing season.

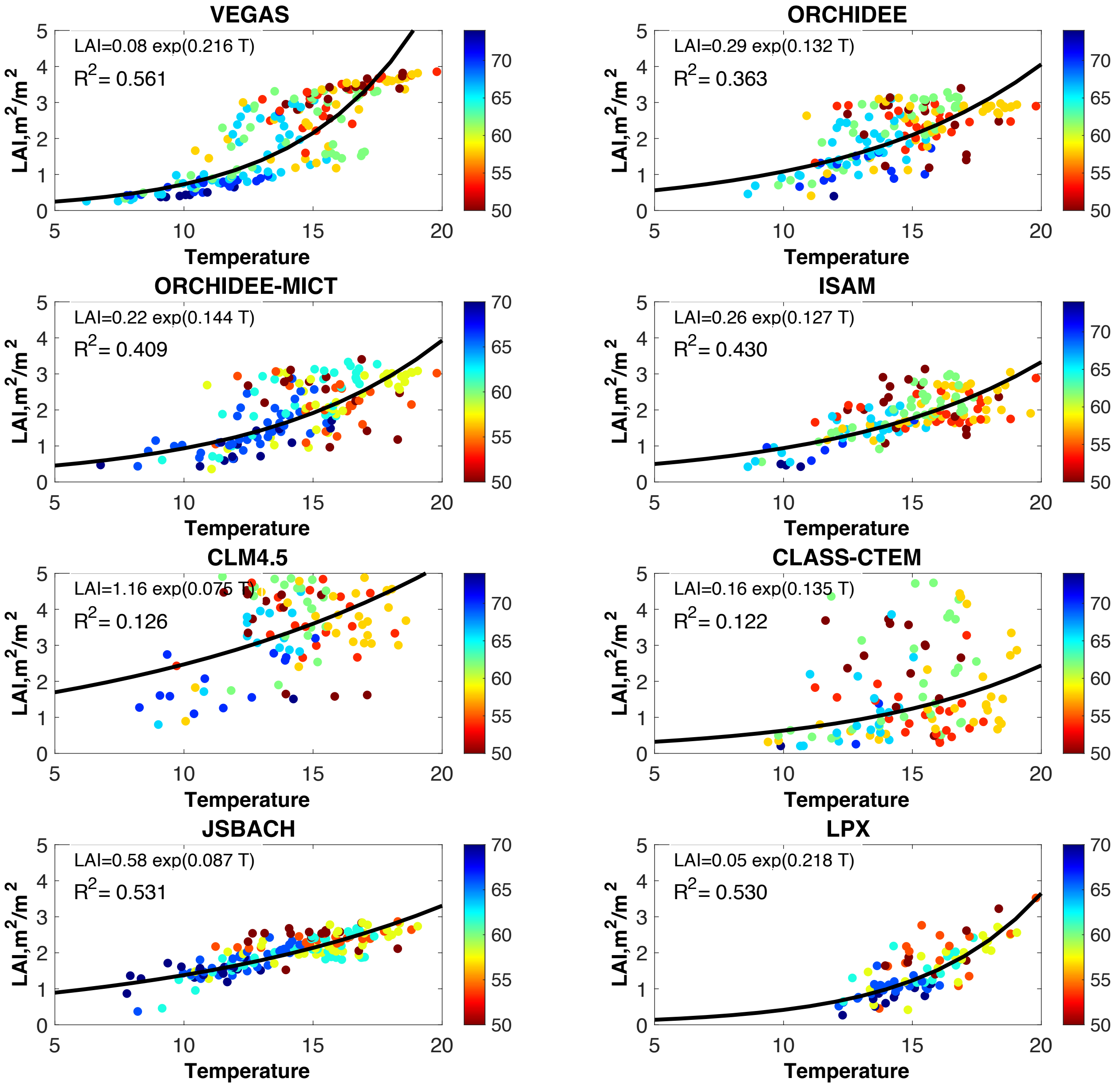

Figure S13 The same as Figure S12, except this is for LAI.

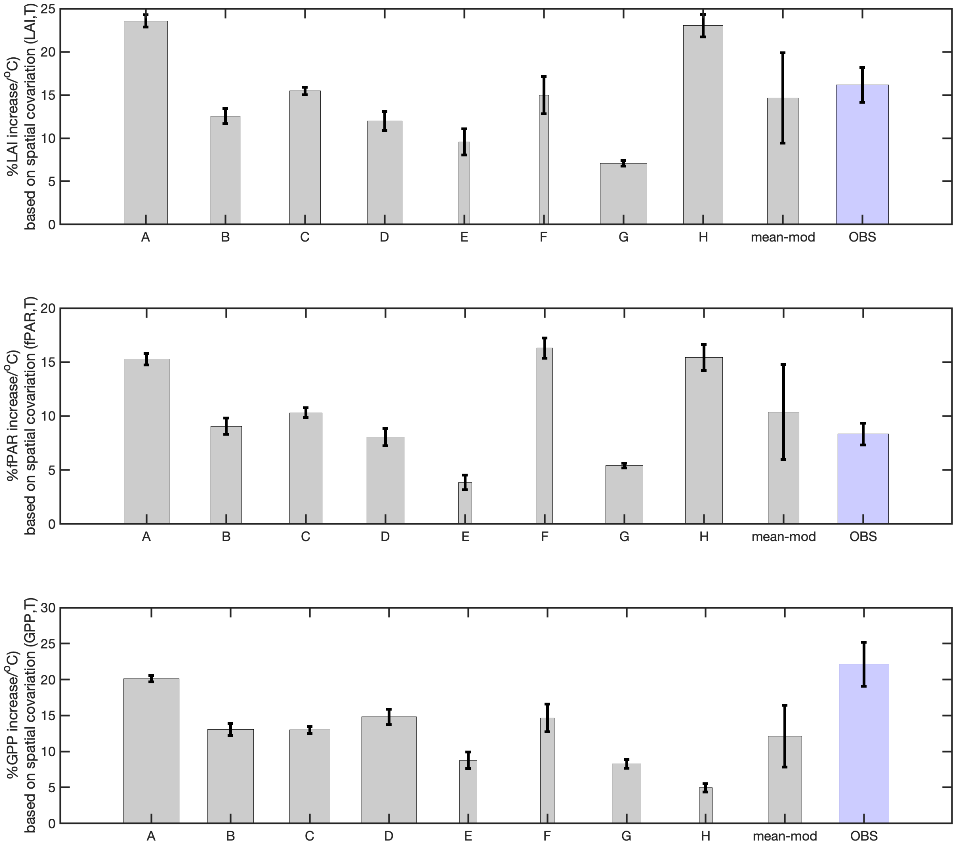

Figure S14 Same as Figure 1 in the main text, except that all models use the condensed MODIS tree cover fraction.

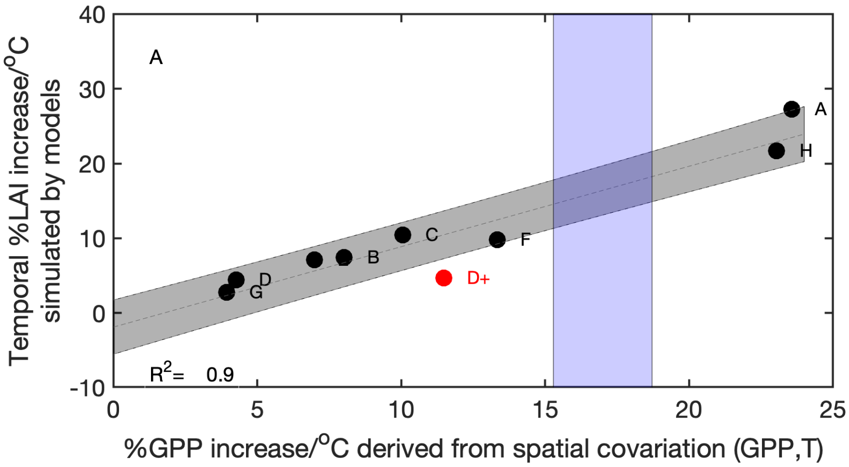

Figure S15 A. The relationship between the spatially-derived and the temporally-derived sensitivities of LAI to temperature in the simulations (γ_{LAI}^{G}). Different from Figure 2A in the main text, we added model D with tundra included (labeled as D+). Model D simulates time-invariant LAI over tundra.

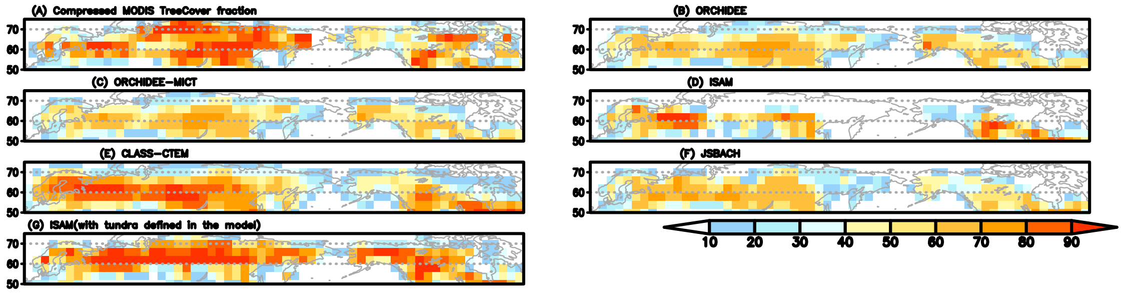

Figure S16 Tree cover fraction between 50°N-75°N (unit:%). (A) Condensed MODIS tree cover fraction that includes needle leaf, broadleaf and shrubland; (B) ORCHIDEE (model B in the main text); (C) ORCHIDEE-MICT (model C); (D) ISAM (model D); € CLASS-CTEM (model F); (F) JSBAC (model G); (G) ISAM including tundra defined in the model;

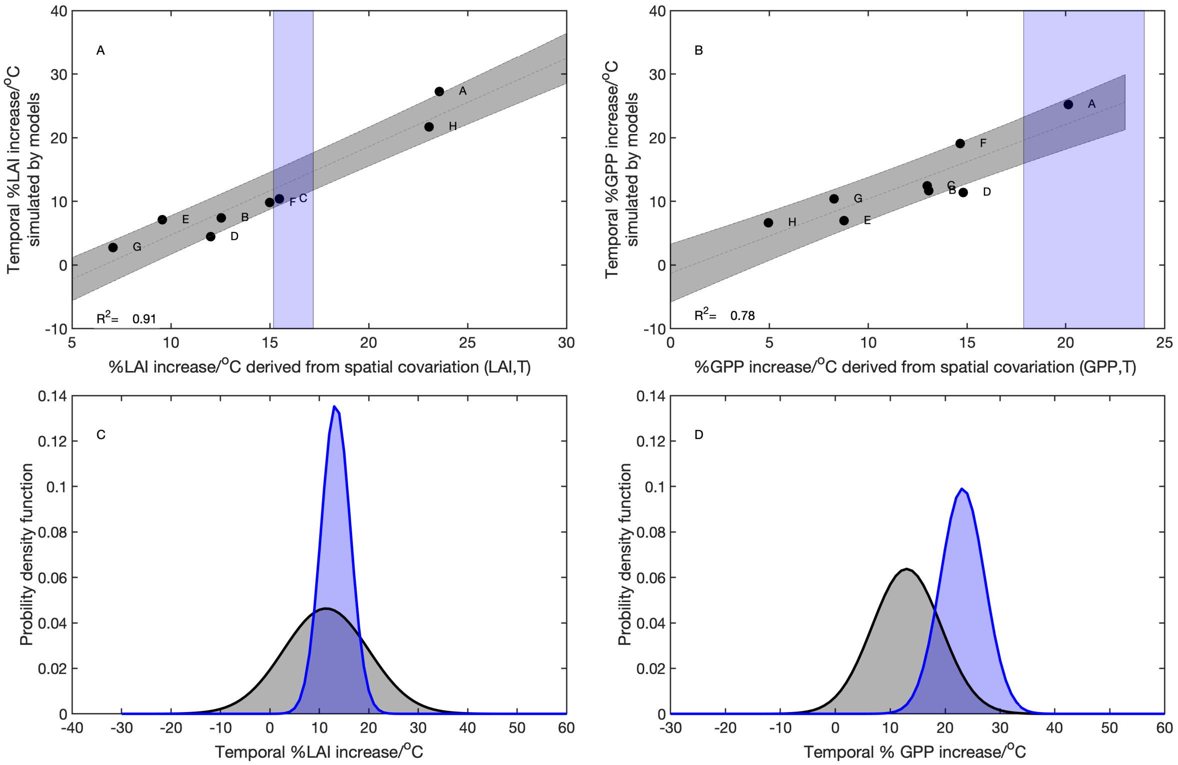

Figure S17 Same as Figure 2 in the main text, except that all the models use the same treecover mask.

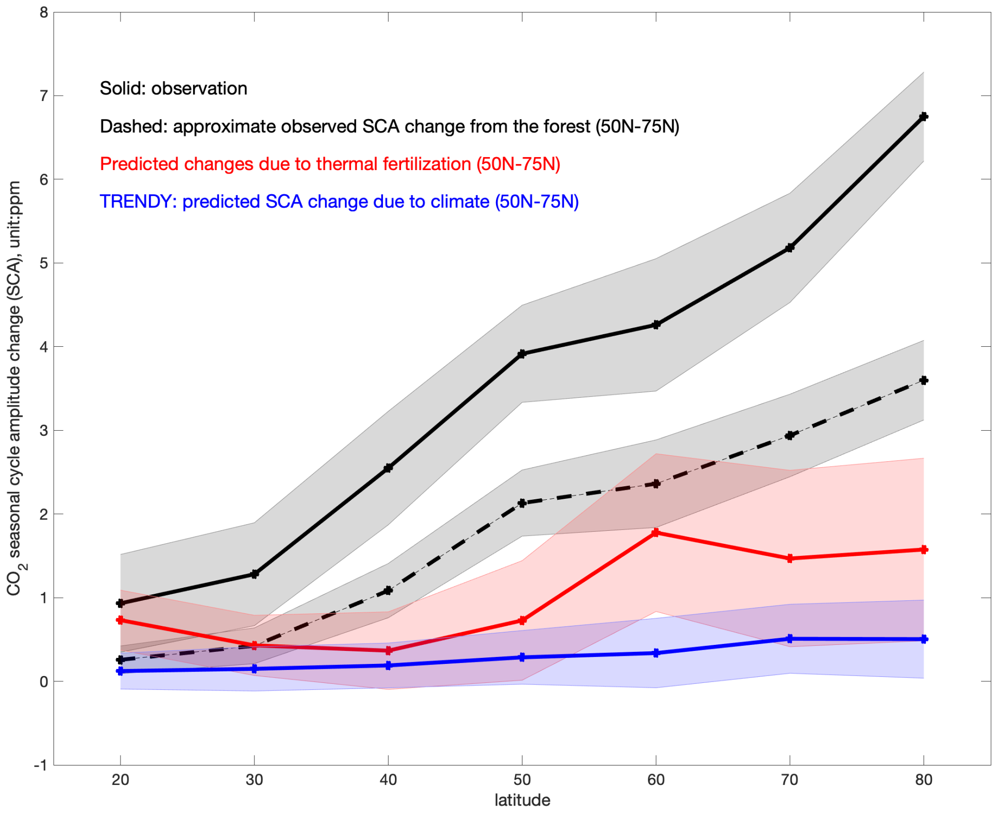

Figure S18 Temperature driven change of NH CO_2 seasonal cycle amplitude. Solid black line: the observed CO_2 seasonal cycle amplitude (SCA) changes between IGY (1958–1963) and HIPPO (2009–2011) aircraft campaigns at 500 hPa. Dashed black line: the approximate CO_2 SCA change between IGY and HIPPO at 500 hPa attributed to the HLNF, which is the multiplication between observed CO_2 SCA and the ratio between model simulated CO_2 SCA forced by forest NEE over 50–75°N and the observed CO_2 SCA (Liu et al., 2020). Red: the calculated CO_2 SCA changes due to the temperature increase of the HLNF. Blue: The simulated CO_2 SCA changes due to climate using the TRENDY S1 and S2 runs. The grey and red lines are the same as Figure 4 in Liu et al., 2020.

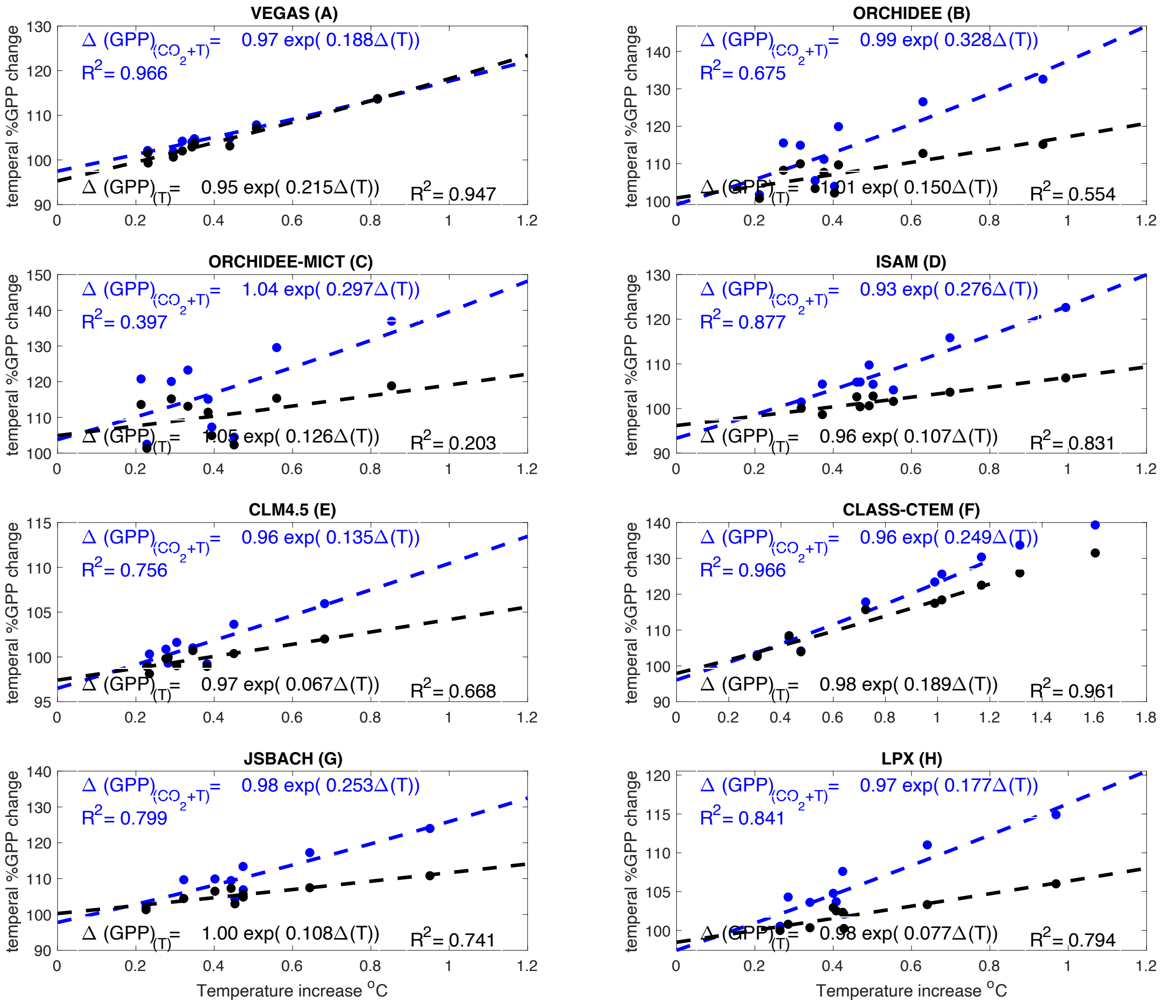

Figure S19 The relationships between temporal percentage change of GPP and temperature increase due to CO_2 and climate effect (blue) and climate effect only (black) for each of the selected TRENDY models. *n* is from 2 to 10. $\Delta GPP_{(CO2+T)}$, $\Delta GPP_{(T)}$, and $\frac{1}{m}\sum_{i=1}^{m}(T_{n,i} - T_{n,0})$ are defined in section 3 in Method.

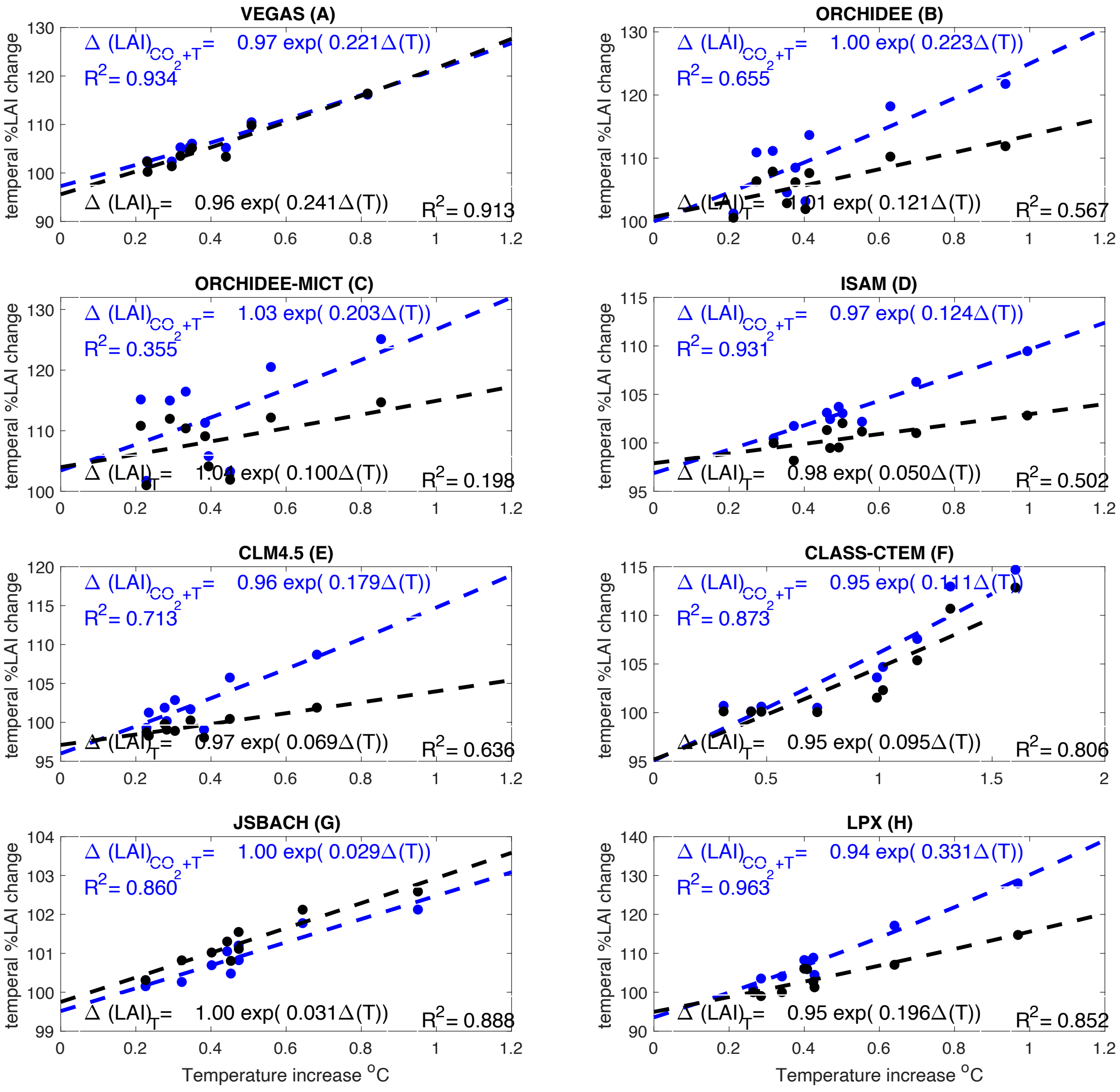

Figure S20 The same as the Figure S19, except this is for LAI.

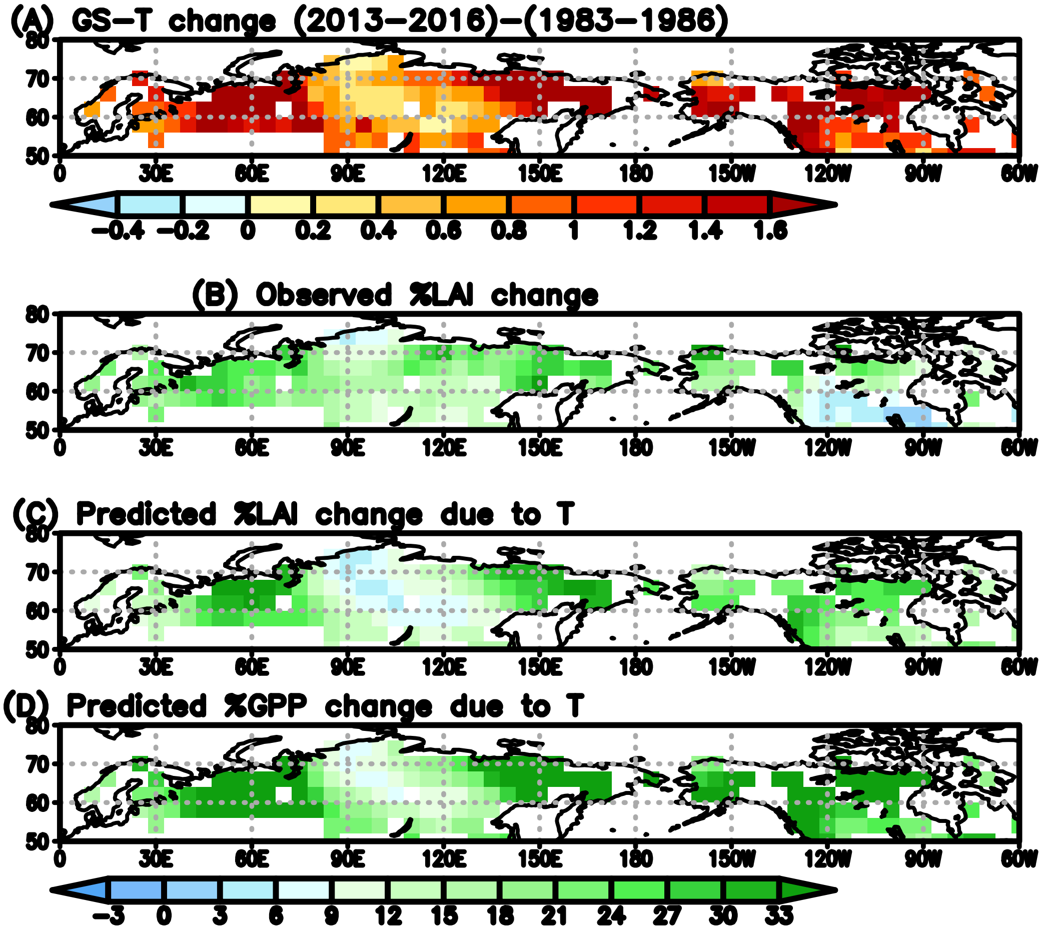

Figure S21 The observationally-constrained LAI- GPP- climate feedback factors γ_{LAI}^{obs} and γ_{GPP}^{obs} imply that temperature increase between 1983-1986 and 2013-2016 contributed to a 9-27% increase in LAI and 12-35% increase in GPP over most of the region. A. The growing season mean temperature change between 1983-1992 and 2006-2015 and (temperature data is derived from the ERA-5 reanalysis); B. Percentage change of LAI due to temperature increase calculated from γ_{LAI}^{obs} ; C. Percentage change of GPP due to temperature increase calculated from γ_{GPP}^{obs}.

Table S1. Summary of TRENDY v6 models. Only models that have monthly GPP and LAI from both S1 and S2 runs, and either R^2(LAI, T) or R^2(GPP, T) larger than 0.2 were selected. For models without land cover fraction information, the compressed IGBP data from MODIS was used to identify grid with at least 40% tree cover.

	ID	R^2(LAI,T)	R^2(GPP, T)	Selected or not	Land Cover	Reference
VEGAS	A	0.7	0.7	Yes	MODIS	Zeng et al., 2005 [52]
ORCHIDEE	B	0.3	0.6	Yes	ORCHIDEE	Krinner et al., 2005[53]
ORCHIDEE-MICT	C	0.3	0.4	Yes	ORCHIDEE-MICT	Guimberteau et al., 2017[54]
ISAM	D	0.1	0.7	Yes	ISAM	Jain et al., 2013[55]
CLM4.5	E	0.2	0.2	Yes	MODIS	Oleson et al., 2013
CLASS-CTEM	F	0.1	0.1	Yes	CLASS-CTEM	Melton et al., 2016[56]
JSBACH	G	0.01	0.3	Yes	JSBACH	Reick et al., 2013[57]
LPX	H	0.6	0.2	Yes	MODIS	Keller et al., 2017[58]
JULES	I	0.0	0.0	R^2(LAI,T))=0.0	JULES	Clark et al., 2011[59]
LPJ-GUESS	J	0.0	0.1	R^2(LAI,T)=0.0	LPJ-GUESS	Smith et al., 2014[60]
VISIT	K	0.0	0.0	R^2(GPP,T)=0.0	VISIT	Kato et al., 2013[61]
CABLE	N/A	N/A	N/A	No S2 run	N/A	Haverd et al., 2017[62]
DLEM	N/A	N/A	N/A	No LAI	N/A	Tian et al., 2015[63]
